# Bioinspired, Carbohydrate-Containing Polymers Efficiently and Reversibly Sequester Heavy Metals

**DOI:** 10.1021/acscentsci.4c01010

**Published:** 2024-09-11

**Authors:** Sungjin Jeon, Teron Haynie, Samuel Chung, Cassandra E. Callmann

**Affiliations:** Department of Chemistry, The University of Texas at Austin, Austin, Texas 78712, United States

## Abstract

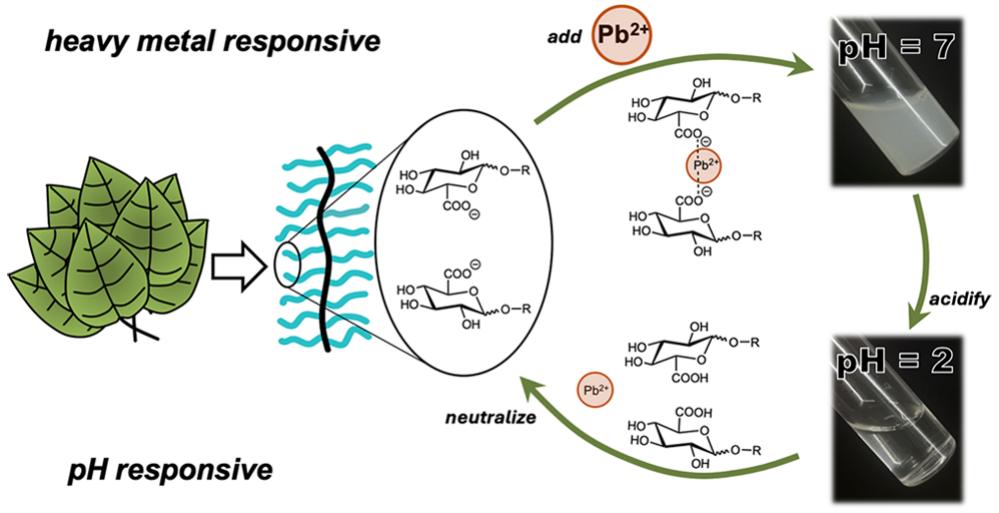

Water scarcity and heavy metal pollution are significant challenges in today’s industrialized world. Conventional heavy metal remediation methods are often inefficient and energy-intensive, and produce chemical sludge. To address these issues, we developed a bioinspired, carbohydrate-containing polymer system for efficient and selective heavy metal removal. Using ring opening metathesis polymerization, we synthesized polymers bearing amphiphilic glucuronate side chains capable of selectively binding heavy metal cations in mixed media. In samples containing high concentrations of heavy metals (>550 ppb), these polymers rapidly form a filterable precipitate upon metal capture, reducing the concentration of cation to <1.5 ppb within 3 min, as measured by inductively coupled plasma mass spectrometry. This system effectively removes cadmium ions from highly contaminated solutions to levels below the Agency for Toxic Substances and Disease Registry limit for Cd^2+^ in drinking water and selectively removes both Cd^2+^ and Pb^2+^ from lake water spiked with trace amounts of metal. Acidification triggers protonation of the glucuronate groups, releasing the heavy metals and resolubilizing the polymer. This capture-and-release process can be repeated over multiple cycles without loss of binding capacity. As such, this study introduces a novel class of recyclable materials with pH-responsive properties, offering potential for applications in water remediation and beyond.

## Abstract 2

A novel carbohydrate-based polymer removes heavy metals from water, forming a recyclable, pH-responsive precipitate. It is highly efficient, needs no carrier matrix, and holds promise for water remediation.

## Introduction

Water is a central resource, essential for the sustenance of life and the functioning of ecosystems.^1^ Clean and safe drinking water is a fundamental human right,^2^ impacting not only public health but also various industrial processes, agriculture, and environmental sustainability.^3^ However, the presence of contaminants, especially heavy metal ions, poses a significant threat to water quality and human health.^4,5^ Heavy metals, including divalent cations such as lead (Pb), cadmium (Cd), nickel (Ni), and mercury (Hg), are notorious for their toxicity and persistence in the environment.^6−8^ Even in minute concentrations, these contaminants can lead to severe health issues and ecological disruptions.^9^ Given the accelerating pace of global industrialization and the increasing scarcity of water, the continued development of efficient materials for water remediation is critical.

Current industrial methods for heavy metal removal (e.g., chemical precipitation, membrane filtration, and sorption) face significant drawbacks, including low removal efficiency, substantial energy consumption, complex regeneration processes, and the generation of toxic chemical sludge.^10^ Moreover, many heavy metals form insoluble precipitates, which causes membrane fouling.^11^ As such, new methods for water purification are needed. In this regard, carbohydrates possess several inherent properties that make them well-suited for the task, including their high adsorptive capabilities, potential for regeneration and reuse, and biocompatibility.^12,13^ Indeed, polysaccharide hydrogels have garnered considerable attention in recent years.^14,15^ Such gels demonstrate considerable efficiency in trapping and isolating heavy metals through adsorptive methods.^16−18^ However, many of these systems operate through ion exchange, consequently elevating water hardness.^19^ Moreover, polysaccharide-based heavy metal traps are constrained by their dependency on hydrogel formation, which have poor mechanical properties and often require additional matrices to function.^12,20^

An ideal material for heavy metal sequestration would be biocompatible, have high selectivity for the target ion(s) over innocuous ions, rapidly and efficiently remove heavy metals through either rapid adsorption or complexation, require minimal energy consumption, minimize chemical sludge formation, and be reusable and easy to regenerate. In this vein, bioinspired materials have gained significant interest over the past several years.^21−25^ These materials are molecularly designed to selectively capture specific metal species from solution and offer promising routes to sustainable purification methods.

Motivated by this, we sought herein to develop a bioinspired, carbohydrate-containing system that rapidly precipitates upon heavy metal binding. Inspired by the ability of homogalacturonans within plant cell walls to capture heavy metals,^26^ we synthesized polymers containing pendant glucuronic acids to mimic this dense presentation of negatively charged sugars, using ring opening metathesis polymerization (ROMP). At neutral pH, the polymers are negatively charged, enabling glucuronate moieties to form ionic bonds with cations. Due to the hydrophobic nature of the poly(norbornene) backbone, these materials undergo a hydrophilicity switch upon binding to heavy metal cations, rapidly forming filterable precipitates and efficiently removing over 99% of these toxic species from solution within minutes. Moreover, these materials are responsive to changes in solution pH, enabling reversible capture-and-release of heavy metals. Upon acidification, the side chains become protonated, which liberates the cations back into solution and forms neutral, glucuronic acid species that are water-soluble. Neutralization of the solution reverses this process, forming precipitates once more between glucuronate side chains and heavy metal cations. Taken together, this innovative development has the potential to significantly impact the design of new water treatment methods, based on bioinspired materials that are stimuli responsive and reusable.

## Results and Discussion

### Synthesis and Physical Analysis of Poly(glucuronate) ROMP Polymers

Inspired by the dense presentation of negatively charged uronic acids in plant cell walls and their ability to irreversibly bind divalent cations,^27,28^ we sought to develop a system based on glucuronic acid that relies on charge-switching and forms filterable precipitates upon heavy metal capture. Our reasoning was that at neutral pH, glucuronic acid primarily exists as its negatively charged, conjugate base (p*K*_a_ = 2.93).^29^ This enables it to form ionic bonds with positively charged species, such as heavy metal cations. This binding event effectively neutralizes the carbohydrate. For these studies, we utilized glucuronic acid in place of galacturonic acid due to its tolerance to decarboxylation^30^ and elimination,^31^ owing to the equatorial hydroxyl group at the C4-position. This feature dramatically enhances the stability of the compound.

To facilitate precipitation upon cation capture, we reasoned that we needed to develop polymers with sufficiently hydrophobic backbones to induce rapid aggregation upon neutralization of the glucuronate groups *via* cation binding. Moreover, we hypothesized that maximizing the grafting density of the glucuronic acids within the polymer scaffold using direct, graft-through polymerization would lead to maximally efficient removal of cations from solution. Thus, we utilized ring opening metathesis polymerization (ROMP), as it affords materials with hydrophobic poly(norbornene) backbones. Moreover, it is a controlled polymerization technique whose initiators display high functional group tolerance,^32,33^ such as the alcohol moieties in carbohydrates.

**Figure 1:**
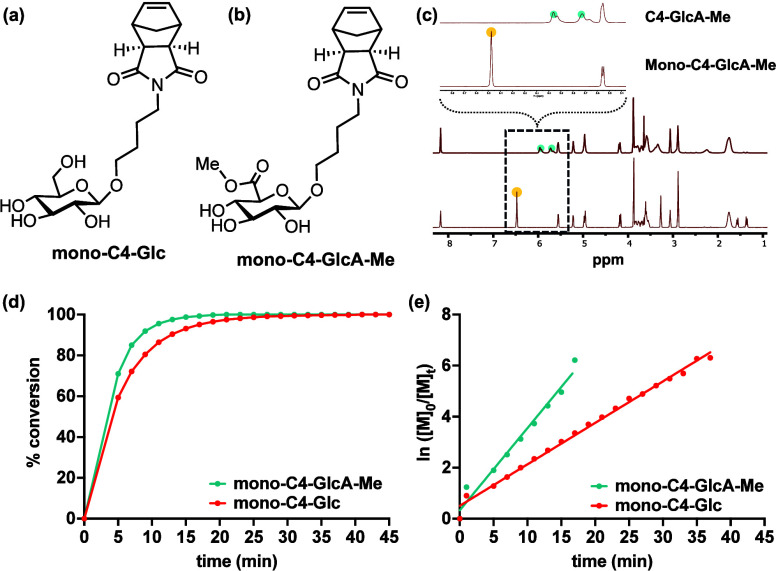
(a) Structure of mono-C4-Glc. (b) Structure of mono-C4-GlcA-Me. (c) ^1^H NMR analysis of the polymerization of mono-C4-GlcA-Me. Disappearance of the monomer alkene peak (yellow) and appearance of two nonequivalent polymer alkene peaks (blue) indicate complete conversion. (d) Conversion of both monomers as a function of time, as measured by ^1^H NMR. (e) Log plots of the conversion of each monomer. Lines represent lines of best fit, using linear least-squares fitting.

With these design considerations in mind, we synthesized ROMP-amenable glucuronic acid derivatives that were linked to norbornenyl moieties through a hydrophobic, 4-carbon alkyl linker (mono-C4-GlcA, Scheme S3). As a control, we synthesized a neutral, glucose-based analogue (mono-C4-Glc, Figure 1a). With synthesized monomers in hand (Scheme S2), we subjected both to graft-through ROMP using Grubbs’ third generation catalyst (Scheme S5). However, mono-C4-GlcA showed no conversion, even in the presence of acid (Figure S1). Therefore, we synthesized a derivative of the monomer containing a methyl-protected carboxylate (mono-C4-GlcA-Me, Figure 1b). We then analyzed the polymerization rate of both the glucose-based monomer (mono-C4-Glc) and protected glucuronic acid monomer (mono-C4-GlcA-Me) using ^1^H NMR (Figure 1c, Figure S3). Both polymerizations were complete within 40 min (Figure 1d), with *k*_obs, Glc_ = 0.16 ± 0.0026 min^–1^ and *k*_obs, GlcA-Me_ = 0.33 ± 0.011 min^–1^, respectively, as determined by linear least-squares fitting (Figure 1e).

**Figure 2:**
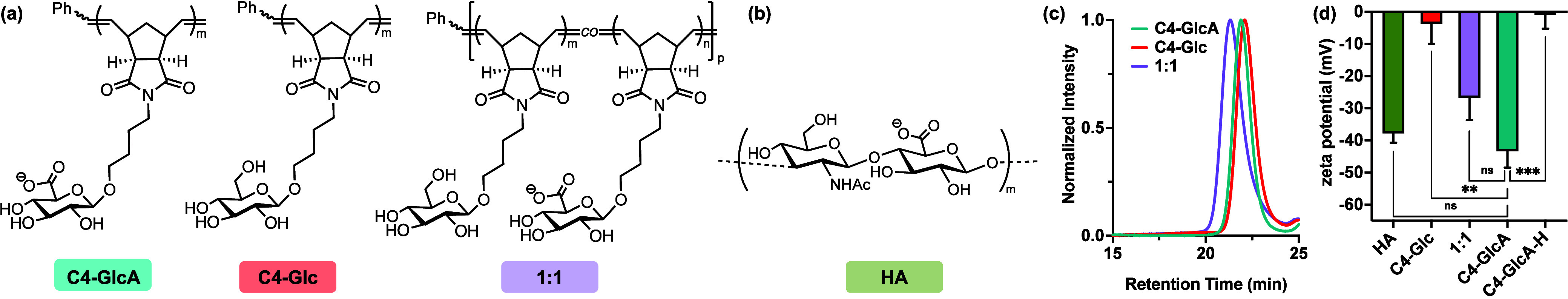
(a) Structure of polymeric C4-GlcA (left), C4-Glc (middle) and 1:1 (right). (b) General structure of commercially available hyaluronic acid (HA). (c) SEC-MALS analysis of C4-GlcA (blue), C4-Glc (red), and 1:1 (purple). (d) Zeta potential of HA (green), C4-Glc (red), 1:1 (purple), C4-GlcA (blue), and C4-GlcA-H (gray). All samples were analyzed at pH = 7.0, except for C4-GlcA-H, which was analyzed at pH = 2.0. Error bars represent the standard deviation of the mean of triplicate samples. Statistical analysis was performed using an ordinary one-way ANOVA, where “**” represents a P value of <0.01 and “***” represents a P value of <0.001.

Upon confirming the ability of both monomers to polymerize, we synthesized three distinct polymers (Figure 2a), one from glucuronic acid (C4-GlcA), one from glucose (C4-Glc), and a 50:50 copolymer of glucose with glucuronic acid (1:1) using ROMP. As a comparison, we performed the same experiments with commercially available hyaluronic acid (HA, Figure 2b), which is a natural polysaccharide that consists of a repeating disaccharide of glucuronic acid with glucosamine. In this way, we could investigate both the influence of carbohydrate charge and grafting density on the binding of divalent cations. We characterized all polymers using size exclusion chromatography with multiangle light scattering (SEC-MALS, Figure 2c) to determine the degree of polymerization (DP) and dispersity (*Đ*). The measured DP closely matched the theoretical DP calculated from the initial monomer-to-initiator ratio ([M]_0_/[I]_0_) for each polymer and all *Đ* were less than 1.20, indicating a controlled polymerization (Table 1). Following polymer analysis, we removed the methyl group from C4-GlcA-Me using lithium hydroxide in a combination of water and tetrahydrofuran (Scheme S6). This afforded a fully deprotected polymer, as confirmed by ^1^H NMR (Figures S4–S5).

**Table 1:** Polymer Characterization by SEC-MALS

polymer	target DP	theo. *M*_n_ (kDa)	*M*_n_ (kDa)	*M*_W_ (kDa)	DP^a^	Đ
C4-Glc	50	19.85	17.43	17.65	44	1.013
C4-GlcA^b^	50	21.25	17.66	17.87	42	1.012
1:1	50	20.25	20.28	23.38	49	1.153

a DP calculated as *M*_n_/MW_monomer_.

b Analyzed using methyl-protected C4-GlcA (C4-GlcA-Me).

We evaluated the zeta potential of all polymers to compare the charge density and their colloidal dispersion stability in neutral aqueous solution (Figure 2d). C4-Glc had a nearly neutral zeta potential of −3.73 mV. Conversely, all charged polymers displayed significantly negative zeta potentials: −26.7 mV (1:1), −37.8 mV (HA) and −43.4 mV (C4-GlcA). Lowering the pH of the solution containing C4-GlcA resulted in a polymer with a neutral surface charge (C4-GlcA-H), indicating the ability of C4-GlcA to undergo charge switching as a function of protonation state. In addition, we evaluated the zeta potential of C4-GlcA as a function of solution pH. As expected, the zeta potential decreases as the pH increases from 2 to 6, where it stabilizes at approximately −40 mV (Figure S6).

### Visualization of Aggregation upon Cd^2+^ Binding

**Figure 3:**
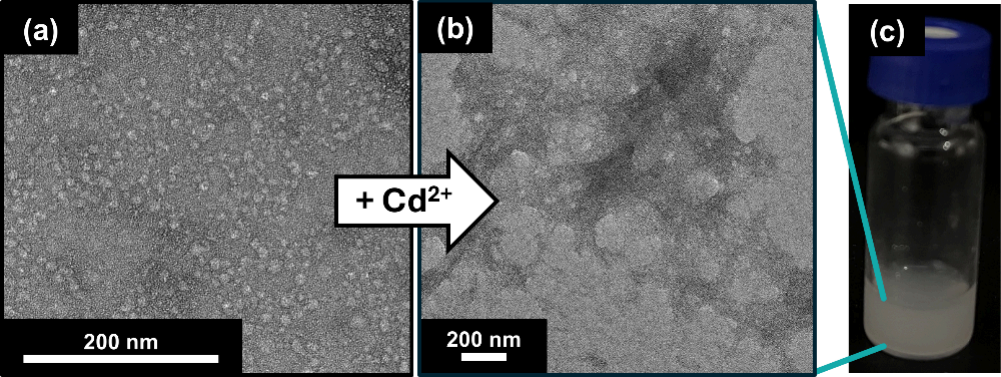
(a) TEM image of C4-GlcA prior to Cd^2+^ addition. (b) TEM image of C4-GlcA after addition of 100 μM Cd^2+^. (c) Precipitate formation upon Cd^2+^ binding by C4-GlcA is visualizable by eye.

As a proof-of-concept, we investigated the interaction between C4-GlcA and Cd^2+^ ions to understand the formation of precipitates and solubility. Prior to Cd^2+^ addition, C4-GlcA forms nanoparticles with sizes ranging from 10 to 30 nm that are visualizable by transmission electron microscopy (TEM, Figure 3a). This confirmed the nanoparticulate nature of C4-GlcA and its full solubility in water. Significantly, there was no evidence of self-aggregation in the absence of Cd^2+^ ions, indicating that C4-GlcA remains in a state ideally suited for trapping ions in aqueous environments. However, addition of Cd^2+^ (100 μM) to a solution of C4-GlcA (1.0 mg/mL) showed the rapid formation of large precipitates (Figure 3b), which are visualizable by both transmission electron microscopy (TEM) and the naked eye (Figure 3c). This drastic increase in aggregate size upon Cd^2+^ addition provides strong evidence of the efficient binding and trapping capabilities of C4-GlcA.

### Colorimetric Analysis of Cd^2+^ Sequestration

**Figure 4:**
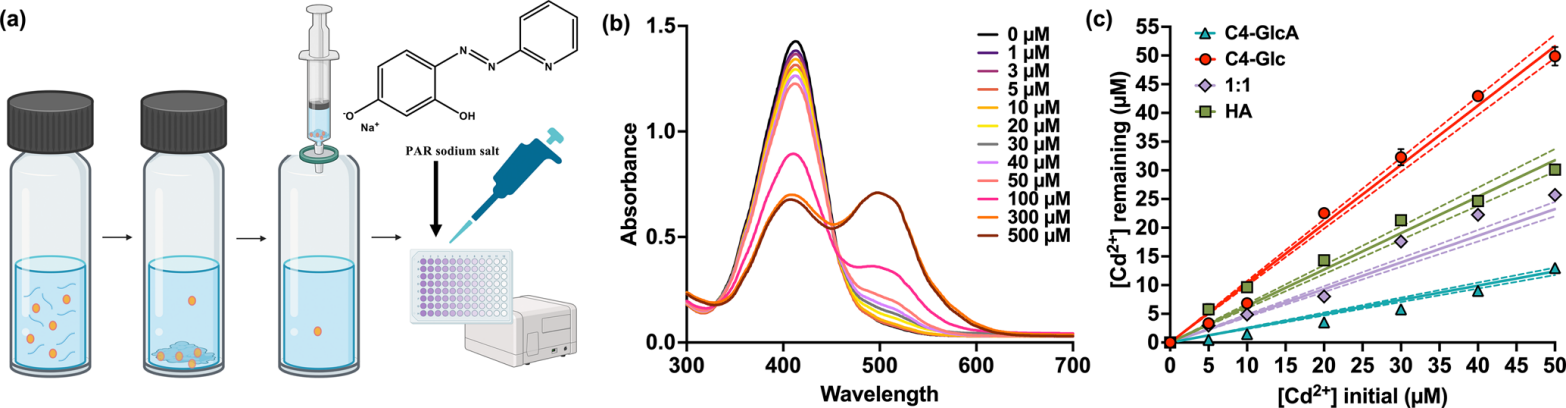
(a) General procedure of the PAR colorimetric dye assay. Cd^2+^ is added to a solution of C4-GlcA (left vial), which rapidly forms precipitates (middle vial). This solution is then filtered into a clean vial (right vial) and added to a 96-well plate, along with the PAR dye. (b) Absorbance of PAR dye when mixed with C4-GlcA that has been incubated with increasing concentrations of Cd^2+^. (c) Concentration of Cd^2+^ remaining in solution after incubation with C4-GlcA (blue), C4-Glc (red), 1:1 (purple), or HA (green). Error bars represent standard deviation (SD) of the mean of triplicate samples. Lines of best fit were calculated using a simple linear regression in GraphPad Prism. Dashed lines represent the 95% confidence interval for each line of best fit.

We initially assessed the ability of C4-GlcA to bind divalent heavy metal cations by developing a colorimetric assay based on 4-(2-Pyridylazo)resorcinol (PAR). This dye is a chromogenic chelator used to determine the concentration of metal ions.^34^ For these analyses, we utilized Cd^2+^, a heavy metal that is known for its lethal impact on aquatic ecosystems.^35^ We first incubated each polymer (0.1 mg/mL C4-GlcA, C4-Glc, 1:1, and HA) with increasing concentrations Cd^2+^ (0 to 500 μM) for 3 min, followed by filtration using a commercially available syringe filter (220 nm). Subsequently, we added the PAR dye to compare the remaining Cd^2+^ content in the filtered solution of each polymer (Figure 4a). We also evaluated a blank solution, which contains only PAR and Cd^2+^.

Before addition of cadmium, the PAR colorimetric dye exhibited an absorption maximum at 413 nm. Upon the addition of Cd^2+^, a gradual increase in the absorption at 496 nm and concurrent decrease at 413 nm was observed for all systems (Figure 4b, Figure S7, Table S1). By keeping the polymer concentration constant across all groups, this analysis enabled us to compare the ability of each system to remove Cd^2+^ from solution (Figure S7). At every Cd^2+^ concentration tested, C4-GlcA was able to remove the most metal from solution (Figure 4c). As expected, C4-Glc was unable to remove Cd^2+^ from solution and matched the blank at all concentrations tested. Interestingly, 1:1 was generally on par with HA in its ability to remove Cd^2+^ at each concentration tested, as determined by unpaired *t* test (Table S2). However, both were significantly worse at removing Cd^2+^ from solution than C4-GlcA at each measured concentration (Table S3). This indicates that maximizing charge density is required to remove the maximum amount of heavy metal.

### Quantitative Analysis of Heavy Metal Removal

**Figure 5:**
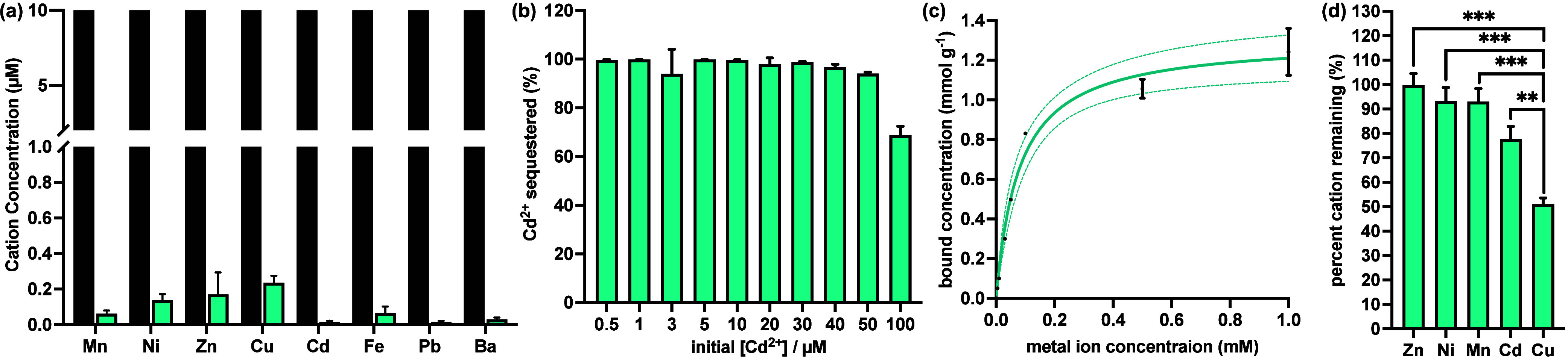
(a) ICP-MS data of the concentration of various cations before (black bars) and after (blue bars) the addition of 0.1 mg/mL C4-GlcA. (b) Amount of Cd^2+^ sequestered by C4-GlcA, as determined by ICP-MS, at increasing concentration of Cd^2+^. (c) Binding isotherm of Cd^2+^ by C4-GlcA. *R*^2^ value = 0.99, as fit by the Langmuir model in GraphPad Prism. Dashed lines represent the 95% confidence interval for the fit. (d) Competitive binding assay, measuring the amounts of various cations that C4-GlcA is able to remove from a single mixed medium containing high (100 μM) concentrations of each cation. Error bars represent the standard deviation (SD) of the mean of triplicate samples. Statistical analysis was performed using an ordinary one-way ANOVA, where “**” represents a *P* value of <0.01, and “***” represents a *P* value of <0.001.

**Figure 6:**
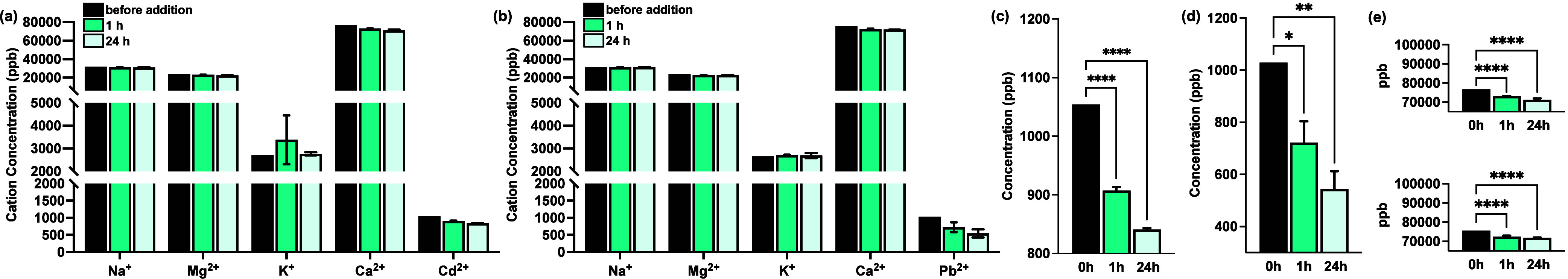
(a) ICP-MS data of concentration of various ions in a sample of the Colorado River that had been spiked with Cd^2+^ before addition of C4-GlcA (black bars), after 1 h incubation with C4-GlcA (dark blue bars), or 24 h incubation with C4-GlcA (light blue bars). (b) ICP-MS data of concentration of various ions in a sample of the Colorado river that had been spiked with Pb^2+^ before addition of C4-GlcA (black bars), after 1 h incubation with C4-GlcA (dark blue bars), or 24 h incubation with C4-GlcA (light blue bars). (c) Expanded view of Cd^2+^ sequestration from spiked lake water sample as shown in panel (a), with statistical analysis after 1 and 24 h incubation. (d) Expanded view of Pb^2+^ sequestration from spiked lake water sample as shown in panel (b), with statistical analysis after 1 and 24 h incubation. (e) Expanded view of Ca^2+^ sequestration from Cd^2+^ spiked lake water sample (top) and Pb^2+^ spiked lake water sample (bottom), with statistical analysis after 1 and 24 h incubation. Error bars represent the standard deviation (SD) of the mean of triplicate samples. Statistical analysis was performed using an ordinary one-way ANOVA, where “*” represents a *P* value of <0.05, “**” represents a P value of <0.01, “***” represents a *P* value of <0.001, and “****” represents a P value of <0.0001.

To more quantitatively assess cation binding, we employed inductively coupled plasma mass spectrometry (ICP-MS). We evaluated the ability of C4-GlcA to capture cations, both in isolation and in mixed solutions. To more closely mimic real-world conditions, we conducted our experiments using water containing minimum concentrations of 40 μM Na^+^, 5 μM K^+^, and 3 μM Ca^2+^. First, we scanned the binding ability of C4-GlcA toward various heavy metal cations. To achieve this, we incubated the polymer (0.1 mg/mL) with a solution of each cation (10 μM) for 3 min to allow for aggregate formation. We then removed the precipitated polymer-cation complex using spin-filtration and assessed the quantity of each cation remaining in solution using ICP-MS. Despite the presence of significantly higher concentrations of monovalent ions such as Na^+^ or K^+^, and in the presence of competing divalent cation Ca^2+^ (later shown to bind C4-GlcA, see Figure 6), C4-GlcA exhibited remarkably high binding affinity for various divalent ions, including Mn^2+^, Ni^2+^, Zn^2+^, Cu^2+^, Cd^2+^, Pb^2+^, and Ba^2+^ (Figure 5a). Over 96% of all cations were removed from each solution, with Cd^2+^ showing the highest removal (99.84%). Furthermore, C4-GlcA also demonstrated superior binding efficiency for the trivalent iron ion (Fe^3+^) with 99.36% sequestration. This exceptional binding performance, especially in the presence of a competitive ionic environment, underscores the potential of this material to function as an effective ion binder.

We next quantitatively assessed the binding capacity of C4-GlcA for Cd^2+^ at 0.1 mg/mL polymer by incubating the material with increasing concentrations of Cd^2+^ (Figure 5b). Significantly, the polymer was able to remove more than 94% of Cd^2+^ from solution after only 3 min incubation, even at highly elevated cation concentrations (50 μM). However, beyond this point, a limitation in the binding capacity of the polymer became apparent, as evidenced by a decrease in the proportion of Cd^2+^ ions that could be trapped at 100 μM. Nevertheless, C4-GlcA could still remove over 70% of Cd^2+^ at this concentration. Notably, when C4-GlcA was incubated with Cd^2+^ ions in concentrations ranging from 0 to 10 μM cation, the residual amount reduces to less than 0.0394 μM (4.373 ppb). This level is significantly below the maximum cadmium concentration in drinkable water as established by the Agency for Toxic Substances and Disease Registry (ATSDR).^36^ This result demonstrates the potential of C4-GlcA to efficiently purify high-concentration contaminated water into the realm of drinkable standards.

To fully evaluate the Cd^2+^ binding performance of C4-GlcA, we incubated the polymer (0.1 mg/mL) with increasing concentrations of Cd^2+^ (up to 1 mM) for either 3 min or 1 h and then evaluated the removal capacity *via* ICP-MS (Figure S8). Extending the incubation time before filtration and ICP-MS analysis did not increase the amount of metal removed, indicating that the sequestration process by C4-GlcA is quick and efficient, occurring within the first 3 min. Using this data, we generated a Cd^2+^ binding isotherm (Figure 5c) with the concentration of bound Cd^2+^ (*q*_e_) plotted as a function of metal concentration (*c*). From this, we determined the maximum binding capacity (*Q*) by fitting the isotherm to the Langmuir model using GraphPad Prism, which revealed a binding capacity of 1.31 ± 0.1 mmol/g. This equates to ∼135 mg/g and is corroborated by the linearized Langmuir isotherm^37^ (Figure S9), where the slope of the line is equal to 1/*Q*. These values are within the range of commercial resins^38,39^ and literature reports of bioinspired adsorbents^21,24^ (1.2–2.9 mmol/g, Table S4).

To preliminarily assess the propensity of C4-GlcA to bind various divalent cations in complex media, we generated a mixed solution of heavy metal cations. We excluded divalent ions that form insoluble precipitates with multiple counterions (e.g., Pb^2+^, Ba^2+^) and then made a solution containing the selected ions before adding the polymer (Figure 5d). Based on the data obtained from our quantitative analysis of Cd^2+^ binding, we set the concentration of each ion to 100 μM to ensure that the total salt concentration was above the saturation limit of the polymer at 0.1 mg/mL. This analysis revealed that more than 93% of Mn^2+^, Ni^2+^, and Zn^2+^ remained in solution. In contrast, only 77.5% of Cd^2+^ ions remained. Interestingly, the polymer seems to possess selectivity of Cu^2+^ over other cations tested, with only 51% of its ions remaining, suggesting potential application in the treatment of Cu-related toxicity.

### Removal of Cd^2+^ and Pb^2+^ from Colorado River Water

To probe the applicability of this system in real-life scenarios, we assessed the ability of C4-GlcA to remove Pb^2+^ and Cd^2+^ from spiked samples of water collected from the Colorado River in Austin, Texas. The authentic water sample contained significantly higher concentrations of competitive ions such as Ca^2+^, Na^+^, and Mg^2+^ than the spiked heavy metal. The concentration of these ions were 75-fold, 31-fold, and 23-fold higher, respectively, than the concentration of Pb^2+^ and Cd^2+^ ions added to the sample (Figure 6). Each sample was preincubated with 100 μM of either Cd^2+^ (Figure 6a) or Pb^2+^ (Figure 6b) for 1 h to equilibrate. After 1 h, the solution was filtered to remove any possible precipitates, and then C4-GlcA was added. The solutions containing the spiked samples were then incubated for either 1 or 24 h to allow for sufficient time for precipitation to occur, and then filtered as above and analyzed by ICP-MS.

At both 1 and 24 h, the concentration of innocuous cations (Na^+^, Mg^2+^, K^+^, Ca^2+^) showed <5% reduction in concentration. Conversely, the concentration of Cd^2+^ and Pb^2+^ decreased as a function of time. Within the first hour, approximately 14% of Cd^2+^ was trapped (Figure 6c), and after 24 h, cadmium sequestration reached nearly 20%. Similarly, measurements of Pb^2+^ trapping in the same water source revealed a trapping capacity close to 30% in 1 h and 45% in 24 h (Figure 6d). When comparing the sequestration of these two heavy metals, it was evident that Pb^2+^, being relatively larger in size, exhibited a greater binding propensity than Cd^2+^. This is consistent with what has been observed in other systems.^40^ Interestingly, C4-GlcA also removed a significant amount of Ca^2+^ along with the heavy metals (Figure 6e). This is in contrast with the function of many ion exchange resins, where Ca^2+^ is displaced by heavier metals, leading to increased water hardness.^19^ This means that in addition to removing heavy metal contaminants, these polymers do not contribute to water hardness. Together, this demonstrates that C4-GlcA holds promise for use, even in water laden with various salts commonly found in real-world conditions.

### pH-Responsive Heavy Metal Capture and Release

**Figure 7:**
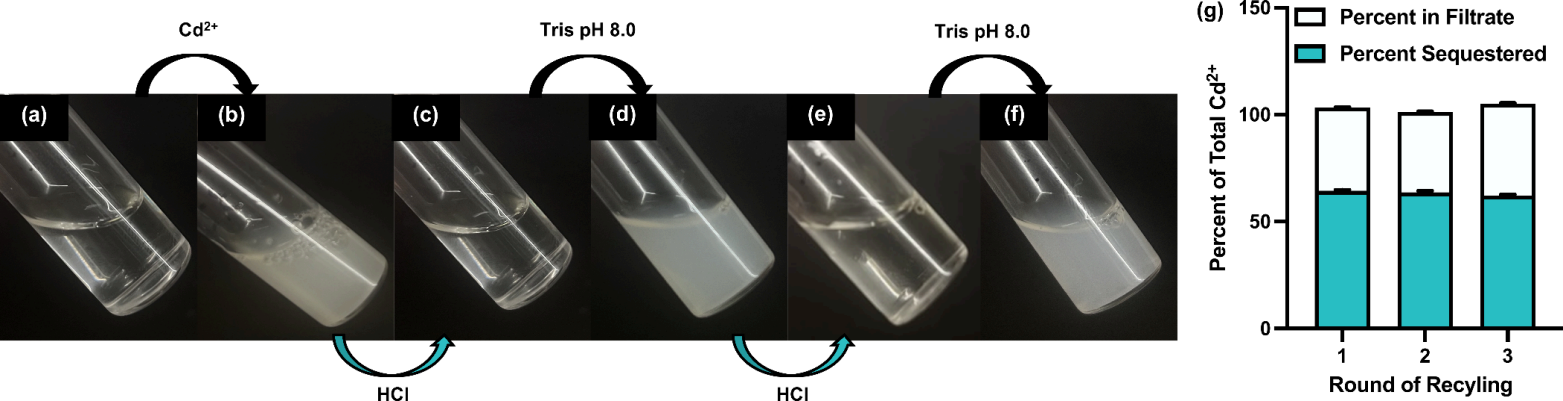
(a) Image of a solution containing 1.0 mg/mL C4-GlcA at neutral pH, prior to addition of Cd^2+^. (b) Upon addition of 10 mM Cd^2+^, rapid precipitation occurs, which is visualizable by eye. (c) Acidification of the solution protonates the C4-GlcA and liberates Cd^2+^ back into solution. This process is repeatable via successive additions of HCl and Tris buffer (d–f). (g) Quantitative analysis of Cd^2+^ sequestration by C4-GlcA across three rounds of recycling. The graph shows the effectiveness of the polymer in removing Cd^2+^ from the solution after each round of recycling. Each bar represents a recycling round, with the blue segment indicating the percentage of Cd^2+^ removed by the polymer and the white segment showing the percentage of Cd^2+^ remaining in the solution. Error bars represent the standard deviation (SD) of the mean of triplicate samples.

Finally, to demonstrate the versatility of C4-GlcA, we qualitatively assessed its ability to capture-and-release heavy metals as a function of solution pH (Video S1). In the experimental setup, we prepared a polymer solution at 1.0 mg/mL (Figure 7a) and added 10 mM of Cd^2+^ ions to induce a visibly detectable level of precipitation. Upon the addition of Cd^2+^, an immediate binding with the polymer occurred, resulting in the formation of a precipitate (Figure 7b). Acidification of the solution *via* HCl addition protonated the carboxylate groups, subsequently releasing the Cd^2+^ ions. Remarkably, even in its protonated state and despite having previously formed dense aggregates, the polymer reentered solution (Figure 7c). We attribute this solubility to the presence of other hydroxyl groups in the glucuronic acid, indicating the polymer’s capacity to revert to its original state. This process is reversible through neutralization with a basic tris buffer (pH = 8). The neutralization of the solution upon the addition of buffer led to the immediate conversion of the polymer back to its carboxylate form, which then rebound the Cd^2+^ ions, forming a precipitate once again (Figure 7d). This cycle was repeated to verify the consistency of the results (Figure 7e–f). We then quantitatively assessed the removal efficiency of C4-GlcA after multiple rounds of recycling, using a polymer concentration of 0.1 mg/mL and a Cd^2+^ concentration of 100 μM. This ensured we were beyond the saturation limit of the polymer, allowing us to observe any differences in sequestration (Figure 7g). Importantly, we observed no loss in sequestration capacity after 3 rounds of recycling, illustrating the pH-responsive nature of C4-GlcA and demonstrating the feasibility of synthesizing a simple, reusable heavy metal trap. The simplicity of the design suggests that this approach could be scalable and economically viable for larger scale applications.

## Conclusion

Given the increasing concern over heavy metal contamination in water sources, the development of a biocompatible, reusable, and efficient method for capturing these contaminants is timely. In this study, we present an innovative approach for water purification using bioinspired, carbohydrate-containing polymers that are specifically designed for heavy metal capture. As a proof-of-concept, we focused on a singular polymer concentration and degree of polymerization. Despite these parameters not being optimized, our findings demonstrate promising results in selectively and efficiently capturing substantial concentrations of Cd^2+^, Pb^2+^, and other heavy metals.

This system offers several advantages over conventional polysaccharide-based heavy metal removal methods. It effectively addresses mechanical strength limitations by forming filterable precipitates without requiring additional matrices. The system requires minimal contact time, achieving removal efficiencies of over 99% within 3 min. The pH-responsive nature allows for easy regeneration and reuse, enabling reversible capture and release of heavy metals simply by adjusting the pH. As such, this system offers the potential to minimize energy consumption and the production of chemical sludge. Moreover, heavy metal capture by this system does not increase water hardness.

Because we utilize ROMP, the synthesis of these materials is highly modular and scalable. As such, there are many handles that can be simultaneously tuned in future endeavors (*e.g*., change degree of polymerization, alter hydrophobicity of backbone) to afford systems with increased binding capacities and selectivities for various metals. Furthermore, owing to their pH-responsive nature, these materials hold promise as easily regenerable and reusable materials for applications in water purification and beyond.

## Supporting Information Available

Materials and methods, synthetic schemes, auxiliary tables, and characterization data (MP4)
Supplemental video showing pH-responsive capture and release (PDF)
