# Supplementary material for: Bioinspired, Carbohydrate-Containing Polymers Efficiently and Reversibly Sequester Heavy Metals
Source: ACS Cent Sci. 2024 Sep 11;10(9):1782–8. doi: 10.1021/acscentsci.4c01010 (PMC11428261; doi:10.1021/acscentsci.4c01010)

SUPPLEMENTAL INFORMATION

for

Bioinspired, Carbohydrate-Containing Polymers Efficiently and Reversibly Sequester Heavy Metals

Sungjin Jeon,[1] Teron Haynie,[1] Samuel Chung[1] and
Cassandra E. Callmann*[1]

[1]Department of Chemistry, University of Texas Austin, Austin, TX 78712, United States.

*Email: ccallmann@utexas.edu

SUPPLEMENTAL INFORMATION
TABLE OF CONTENTS

List of Figures S3

Materials and Methods S4

Monomer Synthesis S6

General Polymerization Procedure S17

Polymerization Kinetics S19

General Procedure for Methyl Ester Deprotection S20

pH-Dependent Zeta-Potential S22

Qualitative Analysis of Cd^{2+} Sequestration S23

ICP-MS Sample Preparation and Analysis S25

TEM Sample Preparation S27

Real Lake Water Sample Preparation S28

Procedure for pH-Dependent Capture-and-Release S29

NMR Spectra S30

SUPPLEMENTAL INFORMATION
LIST OF FIGURES

Figure S1. Polymerization of unprotected glucuronic acid (**C4-GlcA**) monomer S18

Figure S2. Polymerization kinetics of **mono-C4-Glc** and **mono-C4-GlcA-Me** S19

Figure S3. ^{1}H-NMR of **mono-C4-Glc** before and after polymerization S19

Figure S4. ^{1}H-NMR spectra of **1:1** with the deprotection of the methyl ester S21

Figure S5. ^{1}H-NMR spectra of **C4-GlcA-Me** with the deprotection of the methyl ester. S21

Figure S6. pH dependent zeta-potential of **C4-GlcA** S22

Figure S7. Colorimetric assay of PAR as a function of Cd^{2+} concentration S23

Figure S8. Amount of Cd^{2+} remaining by **C4-GlcA**, as determined by ICP-MS S25

Figure S9. Linearized Langmuir isotherm of Cd^{2+} binding by **C4-GlcA** S26

Figure S10. TEM image of acidified **C4-GlcA** S27

Materials

All reagents were of the highest commercial quality and used as received without further purification. Anhydrous dichloromethane (DCM) was obtained from distillation of HPLC grade dichloromethane. Cis-5-norbornene-exo-2,3-dicarboxylic anhydride was obtained from Oakwood chemical. 4-Amino-1-butanol, ethyl β-thioglucopyranoside, D-glucuronic acid lactone, silver trifluoromethanesulfonate were obtained from Ambeed. Anhydrous toluene, 4-methoxybenzyl chloride (PMBCl), sodium hydride 60% dispersion in mineral oil, anhydrous N,N-dimethylformamide (DMF), trimethylsilyl trifluoromethanesulfonate, tert-butyl methyl ether (TBME), glacial acetic acid, anhydrous methanol, HBr in acetic acid, benzyl bromide, 1 M tetrabutylammonium fluoride solution (TBAF), ethyl vinyl ether, sodium chloride, zinc chloride, copper sulfate, iron chloride and amicon ultra – 4 centrifugal filters ultracel - 3K were obtained from sigma-aldrich. Triethylamine, anhydrous pyridine and potassium carbonate were obtained from fisher scientific. N-iodosuccinimide, benzoyl chloride, calcium chloride, manganese chloride and nickel chloride were obtained from Beantown chemical. Magnesium chloride was obtained from VWR. 4-(2-Pyridylazo)resorcinol monosodium salt hydrate was purchased from ThermoScientific chemicals. Colorimetric assays were performed using syringe filter (0.22 µm), which was supplied from ThermoScientific chemicals. Silica flash column chromatography was performed using silica gel (40−63 µm), which was supplied from Sorbtech. Aqueous solutions were freshly prepared with ultra-pure deionized water from a water purification system. Dialysis was performed with Snakeskin dialysis tubing, 3.5K MWCO. 16mm dry I.D. All chemicals not mentioned were obtained from Sigma-Aldrich.

General methods, instrumentation and measurements

Synthetic manipulations that required an inert atmosphere (where noted) were carried out under nitrogen using standard Schlenk techniques. NMR (^{1}H, ^{13}C) spectra were recorded on Bruker Prodigy 500 MHz, Varian 400 MHz and Bruker Advance Neo 400 MHz spectrometer. The ^{1}H, and ^{13}C chemical shifts were reported as δ in units of parts per million (ppm), referenced to the residual solvent. Splitting patterns are denoted as s (singlet), d (doublet), t (triplet), q (quartet), m (multiplet), and br (broad). High-resolution electrospray ionization (ESI) mass spectra were obtained at the mass spectrometry facility (the University of Texas at Austin). Zeta potential was measured by Dynamic Light Scattering Zetasizer Nano ZS. GPC data was measured by using TOSOH EcoSEC Elite HLC-8420GPC, ICP-MS data were obtained by sending samples to Quadrupole ICP-MS lab at the university of Texas at Austin.

Monomer Synthesis

Synthesis of compound **C4-OH**

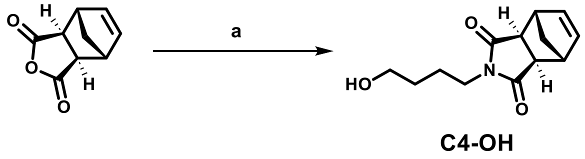

Scheme S1. Synthetic scheme for compounds **C4-OH**, reagents and conditions. (a) 4-amino-1-butanol, dry toluene, reflux, 12 h, 91%.

Compound C4-OH. To a stirred solution of cis-5-norbornene-exo-2,3-dicarboxylic anhydride (5 g, 30.46 mmol) in dry toluene (20 mL) at room temperature under a nitrogen atmosphere was added 4-amino-1-butanol (2.99 g, 33.51 mmol) followed by triethylamine (0.1 mL). After refluxing at 110 ^{o}C for 12 hours, the reaction solvent was removed under reduced pressure. The crude product was purified by column chromatography on silica gel from 20:1 hexanes:ethyl acetate to ethyl acetate as the mobile phase to afford **C4-OH** as a white solid (7.52 g, 91%). ^{1}H-NMR (500 MHz, $CDCl_3$): δ = 6.27 (s, 2H), 3.65 (q, J = 5.9 Hz, 2H), 3.50 (t, J = 7.2 Hz 2H), 3.25 (t, J = 1.9 Hz, 2H), 2.65 (d, J = 1.5 Hz, 2H), 1.60 (m, 4H), 1.49 (d, J = 9.9 Hz, 1H), 1.20 (d, J = 9.9 Hz, 1H). ^{13}C-NMR (126 MHz, $CDCl_3$): δ = 178.20, 137.82, 62.08, 47.80, 45.14, 42.73, 38.39, 29.81, 24.31. HR-MS (ESI): calcd. for $C_{13}H_{17}NO_3$ $[M+Na]^+$ 258.1101, found 258.1111.

Synthesis of compound **mono-C4-Glc**

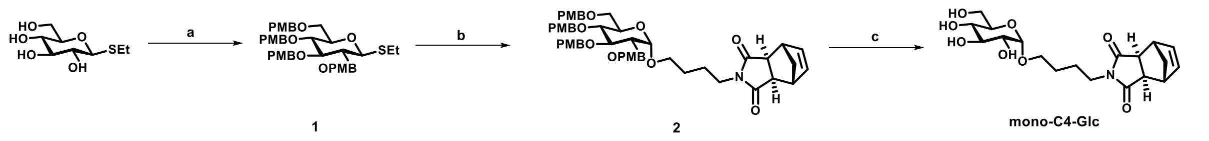

Scheme S2. Synthetic scheme for compounds **mono-C4-Glc**, reagents and conditions. (a) PMBCl, NaH, dry DMF, 0 ºC to RT, 12 h, 82%. (b) **C4-OH**, dry TBME, RT, 3 h, 26% (α yield). (c) 80% AcOH, 70 ºC, 5 h, 88%.

Compound 1. To a stirred solution of Ethyl β-thioglucopyranoside (1 g, 4.462 mmol) in dry DMF (20 mL) was added 60% NaH (0.9 g) at 0 ºC under a nitrogen atmosphere. After stirring for 10 min, PMBCl (3.48 g, 22.31 mmol) was added dropwise to the reaction mixture. The reaction mixture was allowed to warm to room temperature and stirred for 12 hours. The resulting suspension was quenched by the addition of NH_4Cl solution. The mixture was extracted with DCM three times and dried over Na_2SO_4, filtered, and concentrated under reduced pressure. The crude product was purified by column chromatography on silica gel from 20:1 hexanes:ethyl acetate to 3:1 hexanes:ethyl acetate as the mobile phase to afford **1** as a white solid (2.58 g, 82%). ^{1}H-NMR (400 MHz, $CDCl_3$): δ = 7.20 (m, 6H), 7.02 – 6.96 (m, 2H), 6.82 – 6.70 (m, 8H), 4.81 – 4.55 (m, 5H), 4.51 – 4.29 (m, 4H), 3.78 – 3.65 (m, 12H), 3.65 – 3.41 (m, 4H), 3.41 – 3.25 (m, 2H), 2.75 – 2.56 (m, 2H), 1.24 (t, J = 7.4 Hz, 3H). ^{13}C-NMR (100 MHz, $CDCl_3$): δ = 159.37, 159.30, 159.20,

130.86, 130.33, 130.30, 129.98, 129.63, 129.43, 129.37, 113.86, 113.83, 113.81, 113.76, 86.44, 85.11, 81.56, 79.17, 77.79, 77.23, 75.39, 75.12, 74.66, 73.07, 68.79, 55.29, 55.26, 25.01, 15.19. HR-MS (ESI): calcd. for $C_{40}H_{48}O_9S$, $[M+Na]^+$ 727.2911, found 727.2907.

Compound 2. To a stirred solution of **1** (1.5 g, 2.128 mmol) in dry TBME (15 mL) with 3 Å molecular sieves at room temperature under a nitrogen atmosphere was added sequentially **C4-OH** (584 mg, 2.486 mmol) and NIS (700 mg, 3.107 mmol). After stirring for 1 hour, TMSOTf (19 μL, 0.1 mmol) was added dropwise to the reaction mixture. The reaction mixture was stirred for 3 hours. The resulting suspension was quenched by the addition of TEA. The mixture was washed with water three times and dried over Na_2SO_4, filtered, and concentrated under reduced pressure. The crude product was purified by column chromatography on silica gel from 20:1 hexanes:ethyl acetate to 2:1 hexanes:ethyl acetate as the mobile phase to afford **2** as a white solid (485 mg, 26%, α). ^{1}H-NMR (400 MHz, $CDCl_3$): δ = 7.40 – 7.14 (m, 6H), 7.01 (d, J = 8.6 Hz, 2H), 6.94 – 6.69 (m, 8H), 6.35 – 6.22 (m, 2H), 4.89 – 4.25 (m, 9H), 3.87 (m, 1H), 3.84 – 3.72 (m, 12H), 3.70 – 3.34 (m, 9H), 3.30 – 3.21 (m, 2H), 2.66 (d, J = 1.3 Hz, 2H), 1.71 – 1.57 (m, 4H), 1.47 (d, J = 9.8 Hz, 1H), 1.20 (d, J = 9.8 Hz, 1H). ^{13}C-NMR (100 MHz, $CDCl_3$) δ 178.01, 159.34, 159.26, 159.21, 159.14, 137.82, 131.27, 130.56, 130.52, 130.07, 129.63, 129.61, 129.54, 113.85, 113.80, 113.76, 97.09, 81.82, 79.72, 77.44, 77.24, 75.32, 74.66, 73.08, 72.79, 70.20, 68.03, 67.40, 55.29, 55.28, 55.22, 47.83, 45.16, 42.81, 38.40, 26.90, 24.72. HR-MS (ESI): calcd. for $C_{51}H_{59}NO_{12}$ $[M+Na]^+$ 900.3929, found 900.3951.

Compound mono-C4-Glc. Dissolve **2** (400 mg, 0.456 mmol) in 80% AcOH/water solution. The reaction mixture was warmed to 60 ºC and stirred for 5 hours. The reaction mixture was concentrated under reduced pressure. The crude product was purified by column chromatography on silica gel from 20:1 DCM:MeOH to 7:1 DCM:MeOH as the mobile phase to afford **mono-C4-Glc** as a white solid (159 mg, 88%). ^{1}H-NMR (400 MHz, MeOD): δ = 6.22 (t, J = 1.9 Hz, 2H), 4.66 (d, J = 3.8 Hz, 1H), 3.69 (ddd, J = 14.8, 10.6, 4.0 Hz, 2H), 3.59 – 3.49 (m, 2H), 3.49 – 3.32 (m, 4H), 3.27 (dd, J = 9.7, 3.8 Hz, 1H), 3.17 (t, J = 9.4 Hz, 1H), 3.08 (t, J = 1.9 Hz, 2H), 2.62 (d, J = 1.3 Hz, 2H), 1.53 (m, 4H), 1.40 (d, J = 9.9 Hz, 1H), 1.13 (d, J = 9.8 Hz, 1H). ^{13}C-NMR (100 MHz, MeOD): δ = 178.78, 137.51, 98.77, 73.75, 72.33, 72.22, 70.47, 67.02, 61.32, 44.92, 42.13, 37.95, 26.53, 24.33. HR-MS (ESI): calcd. for $C_{19}H_{27}NO_8$ $[M+Na]^+$ 420.1629, found 420.1619.

Synthesis of compound **mono-C4-GlcA**

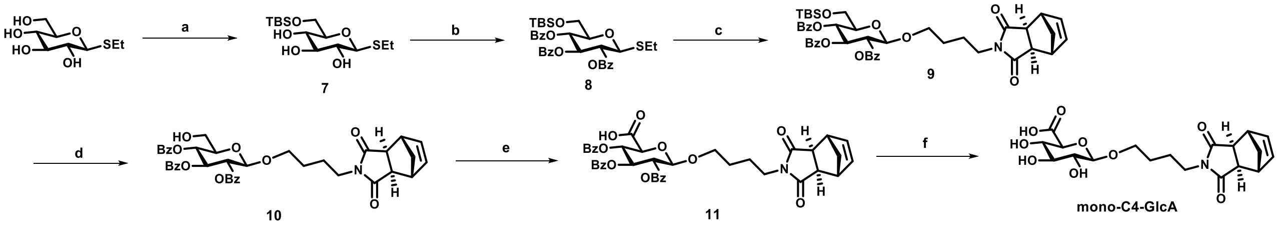

Scheme S3. Synthetic scheme for compounds **mono-C4-GlcA**, reagents and conditions. (a) TBSCl, imidazole, dry DMF, RT, 12 h, 84%. (b) BzCl, dry pyridine, RT, 12 h, 93%. (c) **C4-OH**, TMSOTf, NIS, dry DCM, RT, 4 h. (d) TMSOTf, dry MeOH, RT, 12 h 51% (2 steps). (e) 1) Dess-Martin periodinane, dry DCM, 2) $NaClO_2$, NaH_2PO_4, 2-Me-2-butene, THF:water (1:1), 12 h, 46%. (f) K_2CO_3, dry MeOH, 4 h, 91%.

Compound 7. To a stirred solution of ethyl β-thioglucopyranoside (1 g, 4.462 mmol) in dry DMF (15 mL) at room temperature under a nitrogen atmosphere was added TBSCl (736 mg, 4.910 mmol). After stirring for 12 hours, the mixture was concentrated under reduced pressure. The crude product was extracted with DCM and purified by column chromatography on silica gel from 8:1 hexanes:ethyl acetate to ethyl acetate as the mobile phase to afford **7** as a white solid (1.51 g, 84%).

Compound 8. To a stirred solution of **7** (1.2 g, 3.550 mmol) in dry pyridine (15 mL) at room temperature under a nitrogen atmosphere was added BzCl (8 eq). After stirring for 12 hours, the resulting reaction mixture was concentrated under reduced pressure. The crude product was extracted with DCM and purified by column chromatography on silica gel from 20:1 hexanes:ethyl acetate to 3:1 hexanes:ethyl acetate as the mobile phase to afford **8** as a white solid (2.15 g, 93%).

Compound 9. To a stirred solution of **8** (2.0 g, 3.073 mmol) in dry DCM (15 mL) with 3 Å molecular sieves at room temperature under a nitrogen atmosphere was added sequentially **C4-OH** (794 mg, 3.380 mmol) and NIS (1.04 g, 4.609 mmol). After stirring for 1 hour, TMSOTf (19 μL, 0.1 mmol) was added dropwise to the reaction mixture. The reaction mixture was stirred for 4 hours. The resulting suspension was quenched by the addition of TEA. The mixture was washed with water three times and dried over Na_2SO_4, filtered, and concentrated under reduced pressure. The crude product was used in the next step without further purification.

Compound 10. To a stirred solution of **9** in dry MeOH (15 mL) at room temperature under a nitrogen atmosphere was added TMSOTf (19 μL, 0.1 mmol). The reaction mixture was stirred for 12 hours. The resulting suspension was quenched by the addition of TEA. The mixture was washed with water three times and dried over Na_2SO_4, filtered, and concentrated under reduced pressure. The crude product was extracted with DCM and purified by column chromatography on silica gel from 7:1 hexanes:ethyl acetate to 1:1 hexanes:ethyl acetate as the mobile phase to afford **10** as a white solid (2.18 g, 51%).

Compound 11. To a stirred solution of **10** (1.0 g, 1.410 mmol) in dry DCM (15 mL) at room temperature under a nitrogen atmosphere was added Dess-Martin periodinane (1.20 g, 2.820 mmol). The reaction mixture was stirred for 2 hours. The resulting suspension was filtered through the celite and concentrated under reduced pressure. The crude product was dissolved in THF

followed by the addition of 2-Me-2-butene (100 eq) and mixture of $NaClO_2$ (10 eq), NaH_2PO_4 (10 eq) in water (5 mL). After stirring for 12 hours, the resulting reaction mixture was concentrated under reduced pressure. The crude product was purified by column chromatography on silica gel from ethyl acetate to 20:1 ethyl acetate:MeOH as the mobile phase to afford **11** as a white solid (469 mg, 46%).

Compound mono-C4-GlcA. To a stirred solution of **11** (300 mg, 0.415 mmol) in dry MeOH (15 mL) at room temperature under a nitrogen atmosphere was added K_2CO_3 (140 mg, 1.017 mmol). After stirring for 4 hours, the reaction mixture was filtered by using celite. The crude product was purified by column chromatography on silica gel from 100:1 DCM:MeOH to 4:1 DCM:MeOH as the mobile phase to afford **mono-C4-GlcA** as a white solid (156 mg, 91%). ^{1}H NMR (400 MHz, Acetonitrile-d_3): δ = 6.30 (t, *J* = 1.9 Hz, 2H), 4.27 (d, *J* = 7.7 Hz, 1H), 3.88 – 3.80 (m, 1H), 3.74 (d, *J* = 9.6 Hz, 1H), 3.51 (dt, *J* = 10.5, 6.1 Hz, 1H), 3.43 (td, *J* = 10.0, 8.3, 4.0 Hz, 3H), 3.33 (t, *J* = 9.0 Hz, 1H), 3.17 – 3.10 (m, 3H), 2.66 (d, *J* = 1.5 Hz, 2H), 1.58 (d, *J* = 5.3 Hz, 4H), 1.43 (dt, *J* = 9.8, 1.6 Hz, 1H), 1.22 (d, *J* = 9.8 Hz, 1H). LC-MS (ESI): calcd. for $C_{19}H_{25N}O_9$ $[M+H]^+$ 412.15, found 412.1.

Synthesis of compound **mono-C4-GlcA-Me**

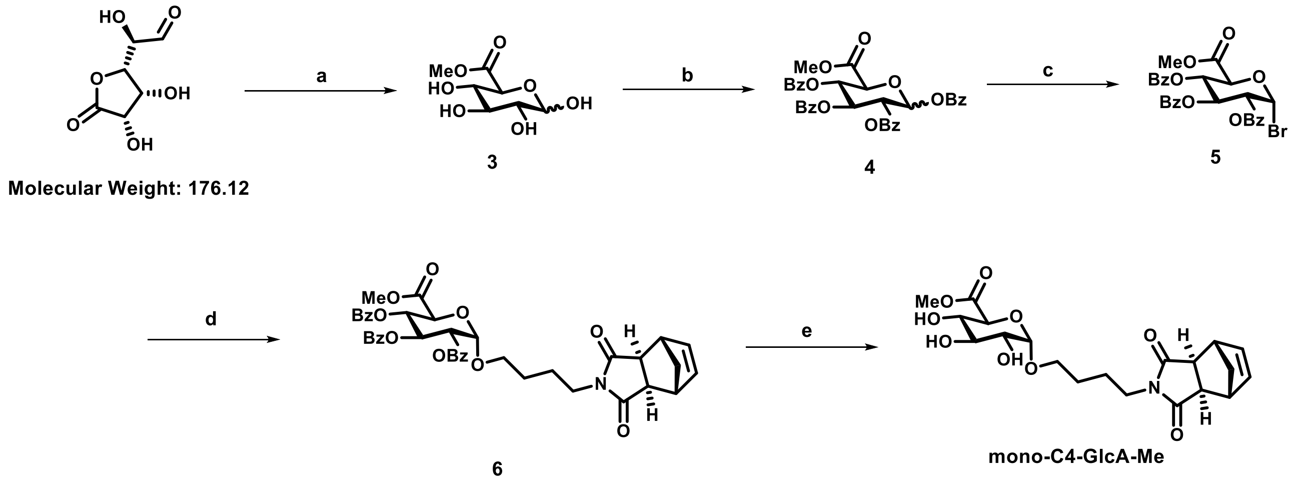

Scheme S4. Synthetic scheme for compounds **mono-C4-GlcA-Me**, reagents and conditions. (a) NaOMe, dry MeOH, RT, 12 h. (b) BzCl, dry pyridine, RT, 12 h, 76% (2 steps). (c) HBr in AcOH, dry DCM, RT, 12 h, 74%. (d) **C4-OH**, AgOTf, dry DCM, RT, 12 h, 46% (α yield). (e) K_2CO_3, dry MeOH, RT, 4 h, 82%

Compound 3. To a stirred solution of D-glucuronic acid lactone (1.5 g, 8.523 mmol) in dry MeOH (15 mL) with 3 Å molecular sieves at room temperature under a nitrogen atmosphere was added NaOMe (23 mg, 0.426 mmol). After stirring for 12 hours, the mixture was concentrated under reduced pressure. The crude product was used in the next step without further purification.

Compound 4. To a stirred solution of 3 in dry pyridine (15 mL) at room temperature under a nitrogen atmosphere was added BzCl (4.97 ml, 42.62 mmol). After stirring for 12 hours, the resulting reaction mixture was concentrated under reduced pressure. The crude product was

extracted with DCM and purified by column chromatography on silica gel from 20:1 hexanes:ethyl acetate to 3:1 hexanes:ethyl acetate as the mobile phase to afford **4** as a white solid (4.04 g, 76%, 2 steps). ^{1}H-NMR (400 MHz, $CDCl_3$): δ = 8.08 – 7.87 (m, 8H), 7.60 – 7.29 (m, 12H), 6.34 (d, J = 6.9 Hz, 1H), 6.02 (t, J = 8.5 Hz, 1H), 5.88 – 5.79 (m, 2H), 4.63 (d, J = 8.4 Hz, 1H), 3.60 (s, 3H). ^{13}C-NMR (100 MHz, $CDCl_3$): δ = 167.13, 165.44, 165.24, 165.00, 164.53, 133.91, 133.61, 133.53, 133.52, 130.26, 129.92, 129.90, 129.89, 128.70, 128.67, 128.53, 128.45, 128.39, 92.07, 73.34, 71.09, 69.43, 52.98.(beta). HR-MS (ESI): calcd. for $C_{35}H_{28}O_{11}$ $[M+Na]^+$ 647.1524, found 647.1521.

Compound 5. To a stirred solution of **4** (2 g, 3.205 mmol) in dry DCM (15 mL) at 0 ºC under a nitrogen atmosphere was added HBr in AcOH (15 mL). The reaction mixture was allowed to warm to room temperature and stirred for 12 hours. The resulting reaction mixture was quenched by sat. $NaHCO_3$ and extracted with DCM. The crude product was purified by column chromatography on silica gel from 20:1 hexanes:ethyl acetate to 2.5:1 hexanes:ethyl acetate as the mobile phase to afford **5** as a white solid (1.38 g, 74%). ^{1}H-NMR (400 MHz, $CDCl_3$): δ = 8.18 – 7.84 (m, 7H), 7.65 – 7.22 (m, 8H), 6.90 (d, J = 4.0 Hz, 1H), 6.28 (t, J = 9.8 Hz, 1H), 5.74 (t, J = 9.9 Hz, 1H), 5.41 – 5.24 (m, 1H), 4.86 (d, J = 10.2 Hz, 1H), 3.69 (s, 3H). ^{13}C-NMR (100 MHz, $CDCl_3$): δ = 166.78, 165.44, 165.26, 165.19, 133.86, 133.68, 133.49, 130.08, 129.92, 129.80, 128.69, 128.60, 128.56, 128.52, 128.44, 128.30, 85.71, 72.52, 71.04, 69.06, 53.16. HR-MS (ESI): calcd. for $C_{28}H_{23}BrO_9$ $[M+Na]^+$ 605.0418, found 605.0423.

Compound 6. To a stirred solution of 5 (1.0 g, 1.718 mmol) in dry DCM (15 mL) with 3 Å molecular sieves at room temperature under a nitrogen atmosphere was added sequentially C4-OH (484 mg, 2.061 mmol). After stirring for 1 hour, AgOTf (883 mg, 3.436 mmol) was added to the reaction mixture. The reaction mixture was stirred for 12 hours. The resulting suspension was filtered by using celite. The mixture was washed with water three times and dried over Na_2SO_4, filtered, and concentrated under reduced pressure. The crude product was purified by column chromatography on silica gel from 20:1 hexanes:ethyl acetate to 1:1 hexanes:ethyl acetate as the mobile phase to afford **6** as a white solid (583 mg, 46%, α). ^{1}H-NMR (400 MHz, $CDCl_3$): δ = 8.01 – 7.83 (m, 6H), 7.56 – 7.24 (m, 9H), 6.27 (t, J = 1.8 Hz, 2H), 6.16 (d, J = 9.8 Hz, 1H), 5.65 (t, J = 9.8 Hz, 1H), 5.42 (d, J = 3.7 Hz, 1H), 5.33 (dd, J = 10.1, 3.7 Hz, 1H), 4.60 (d, J = 10.0 Hz, 1H), 3.85 (m, 1H), 3.69 (s, 3H), 3.55 – 3.40 (m, 3H), 3.27 – 3.15 (m, 2H), 2.67 – 2.52 (m, 2H), 1.72 – 1.53 (m, 4H), 1.47 (d, J = 9.9, 1H), 1.16 (d, J = 9.9 Hz, 1H). ^{13}C-NMR (100 MHz, $CDCl_3$): δ = 177.94, 168.21, 165.62, 165.35, 137.80, 133.41, 133.22, 129.89, 129.74, 129.07, 128.92, 128.91, 128.48, 128.44, 128.33, 96.41, 71.41, 70.22, 69.78, 68.71, 68.64, 52.89, 47.78, 45.14, 42.74, 38.13, 26.76, 24.49. HR-MS (ESI): calcd. for $C_{41}H_{39}NO_{12}$ $[M+Na]^+$ 760.2364, found 760.2362.

Compound mono-C4-GlcA-Me. To a stirred solution of **6** (500 mg, 0.678 mmol) in dry MeOH (15 mL) at room temperature under a nitrogen atmosphere was added K_2CO_3 (140 mg, 1.017 mmol). After stirring for 4 hours, the reaction mixture was filtered by using celite. The crude product was purified by column chromatography on silica gel from 100:1 DCM:MeOH to 9:1 DCM:MeOH as the mobile phase to afford **mono-C4-GlcA-Me** as a white solid (236 mg, 82%). ^{1}H-NMR (400 MHz, MeOD): δ = 6.23 (t, J = 1.9 Hz, 2H), 4.70 (d, J = 3.7 Hz, 1H), 3.96 (d, J =

9.8 Hz, 1H), 3.66 (s, 3H), 3.65 – 3.58 (m, 1H), 3.53 (t, J = 9.3 Hz, 1H), 3.46 – 3.36 (m, 4H), 3.33 (dd, J = 9.6, 3.7 Hz, 1H), 3.09 (t, J = 1.9 Hz, 2H), 2.63 (d, J = 1.4 Hz, 2H), 1.54 (tdd, J = 14.2, 7.3, 4.0 Hz, 4H), 1.40 (d, J = 9.9, 1H), 1.14 (d, J = 9.9 Hz, 1H). ^{13}C-NMR (100 MHz, MeOD): δ = 178.77, 170.57, 137.53, 99.42, 73.10, 71.96, 71.75, 71.62, 67.73, 51.43, 44.93, 42.17, 37.89, 26.53, 24.29. HR-MS (ESI): calcd. for $C_{20}H_{27}NO_9$ $[M+Na]^+$ 448.1578, found 448.1586

General polymerization procedure

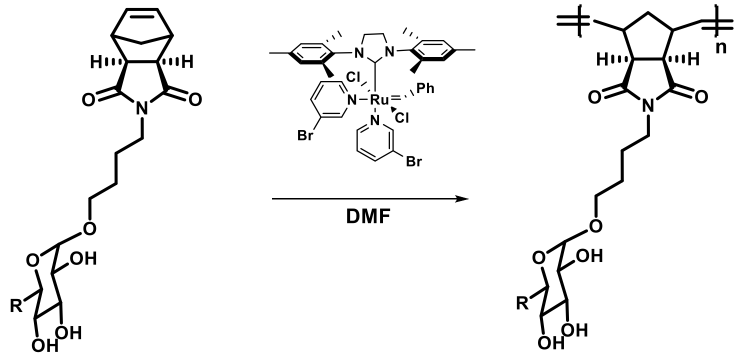

R_1 = CH_2OH
R_2 = COOH
R_3 = COOMe

Scheme S5. General polymerization procedure using graft-through technique.

Grubbs 3rd generation catalyst (1 eq), as calculated, was added to a solution of each monomer (30 mg, 50 eq) in dry DMF (1 mL). After stirring for 1 hour, the reaction mixture was quenched using 20 equivalents of ethyl vinyl ether. The reaction mixture was then precipitated by adding an excess amount of ether and filtered to obtain each polymer.

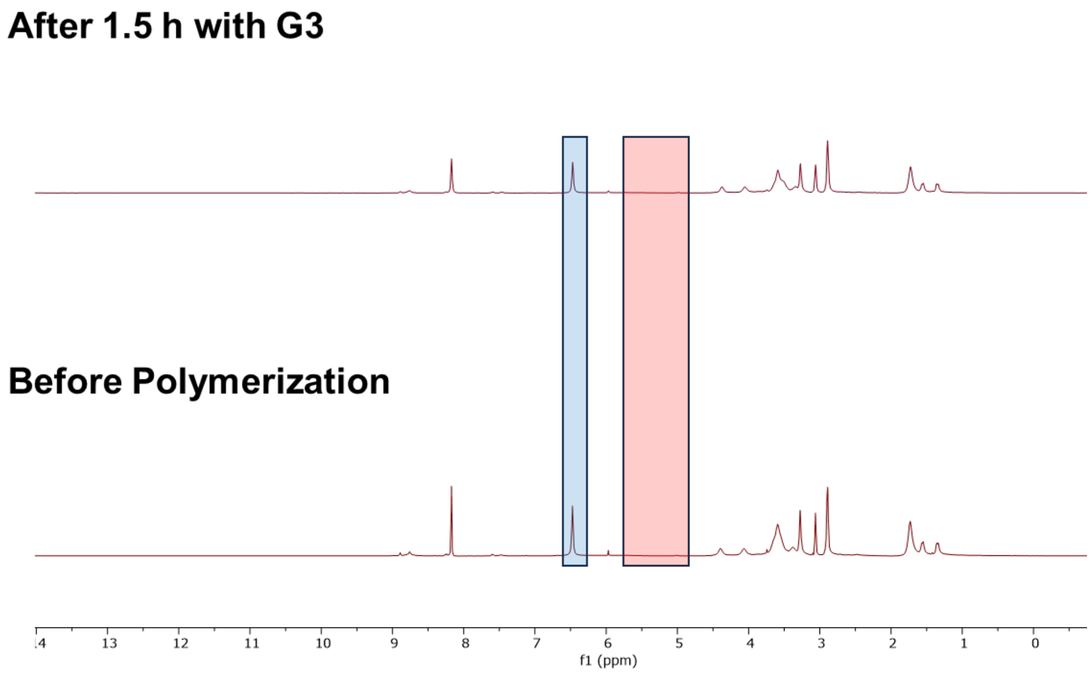

Figure S1. Polymerization of unprotected glucuronic acid (C4-GlcA) monomer.

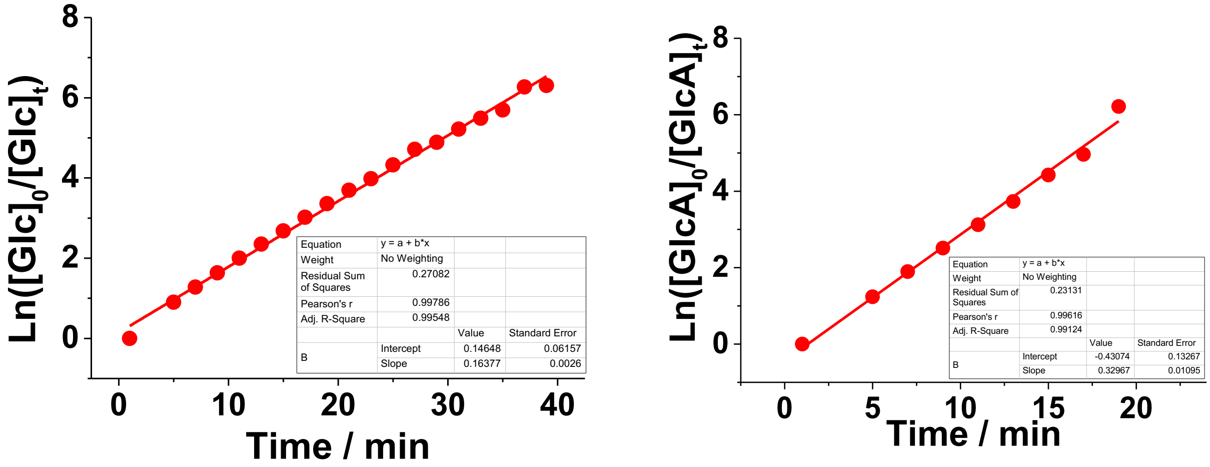

Figure S2. Polymerization kinetics of mono-C4-Glc and mono-C4-GlcA-Me.

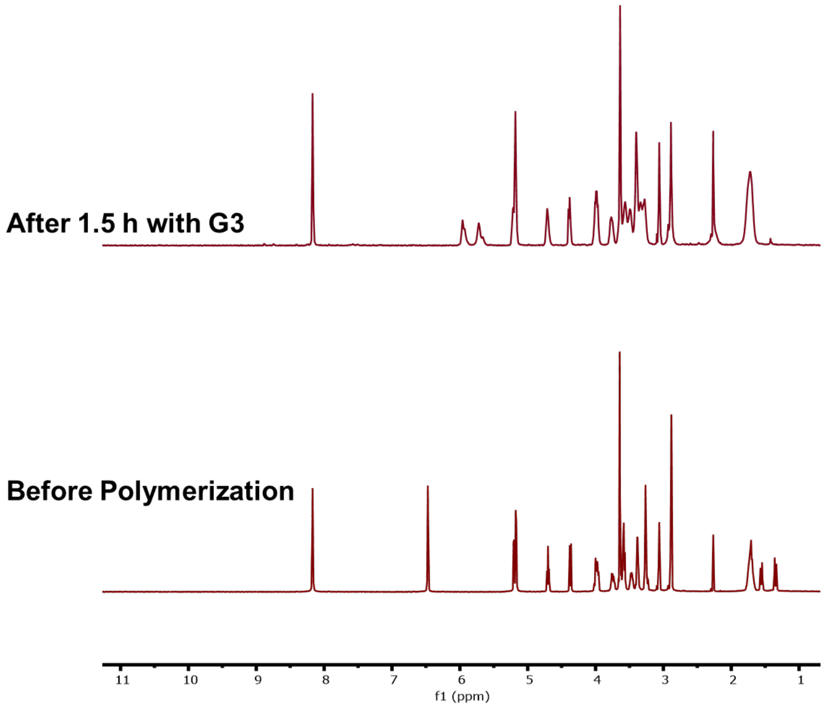

Figure S3. ^{1}H-NMR of mono-C4-Glc before and after polymerization.

General Procedure for Methyl Ester Deprotection

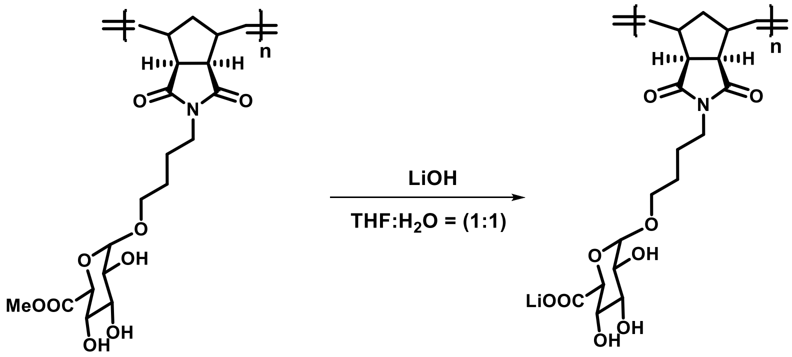

Scheme S6. General deprotection of methyl ester procedure.

Polymers, designated as **1:1**, **C4-GlcA**, were individually prepared for deprotection. Each polymer was initially dissolved in a solvent system comprising a 1:1 ratio of tetrahydrofuran (THF) and water. Subsequently, lithium hydroxide (LiOH) was added to the solution, ensuring a stoichiometric ratio of 2 equivalents (eq) of LiOH per equivalent of the COOMe. This reaction mixture was then agitated continuously for a period of 2 hours to facilitate the deprotection reaction. Following the completion of the reaction, the mixture was subjected to a desalting process. This was accomplished using Snakeskin dialysis tubing, characterized by a molecular weight cut-off (MWCO) of 3.5K. The success and progress of the deprotection process for each polymer were monitored using ^{1}H-NMR spectroscopy.

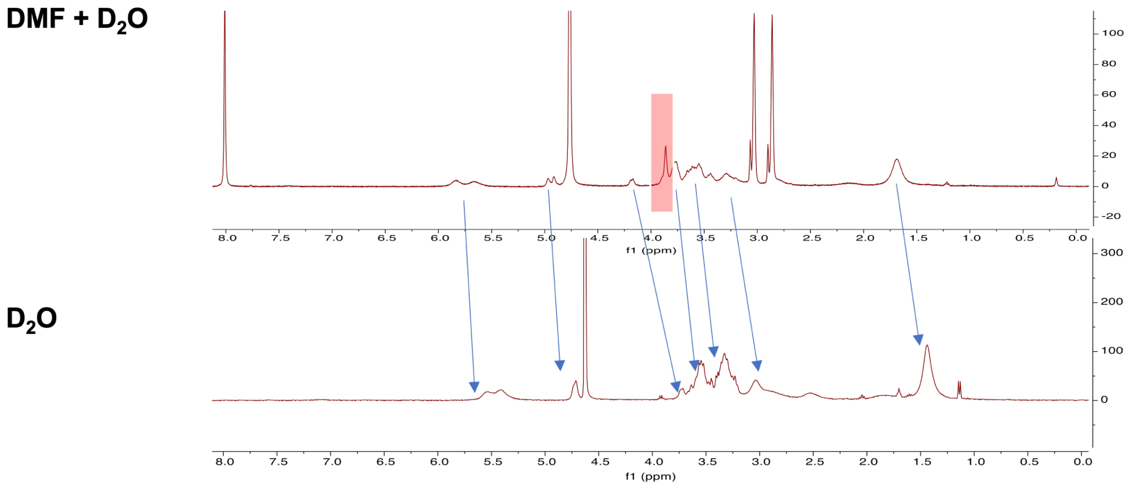

Figure S4. ^{1}H-NMR spectra of **1:1** before and after deprotection of the methyl ester group.

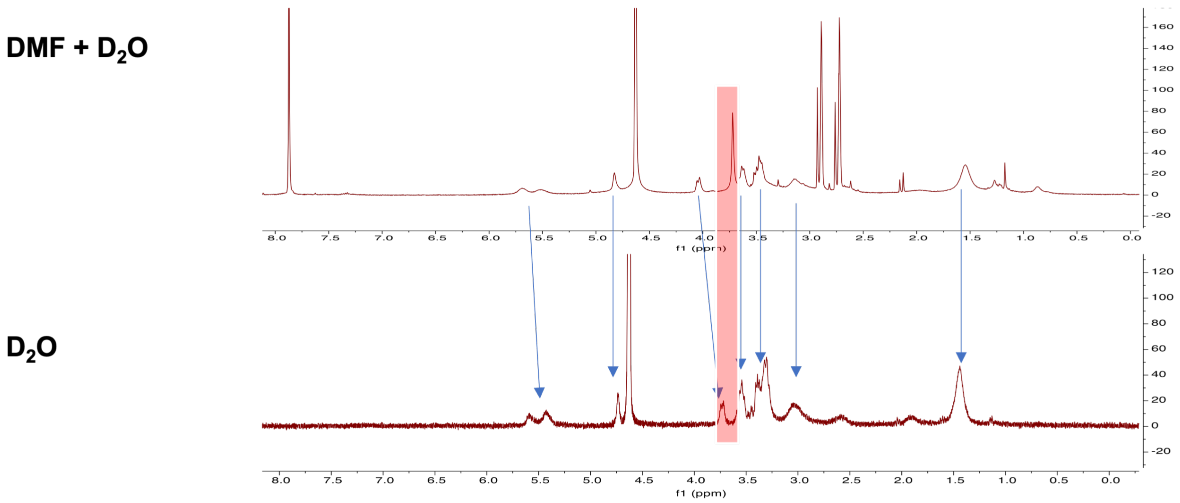

Figure S5. ^{1}H-NMR spectra of **C4-GlcA-Me** with the deprotection of the methyl ester group.

pH-Dependent Zeta-Potential

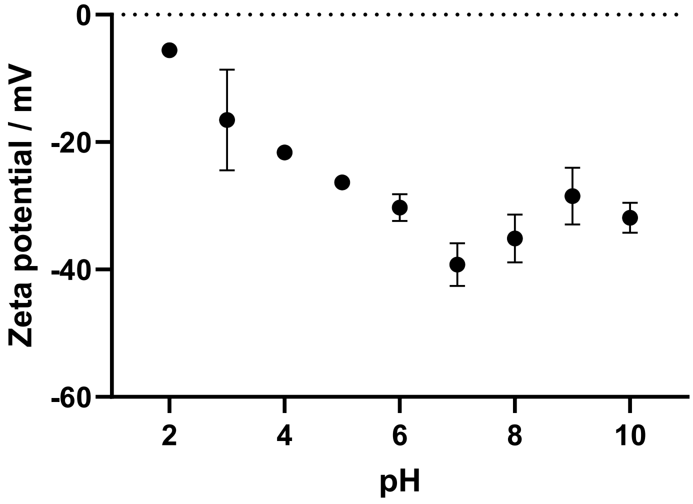

Figure S6. pH dependent zeta-potential of **C4-GlcA**. [**C4-GlcA**] = 0.5 mg/ml.

Qualitative Analysis of Cd^{2+} Sequestration

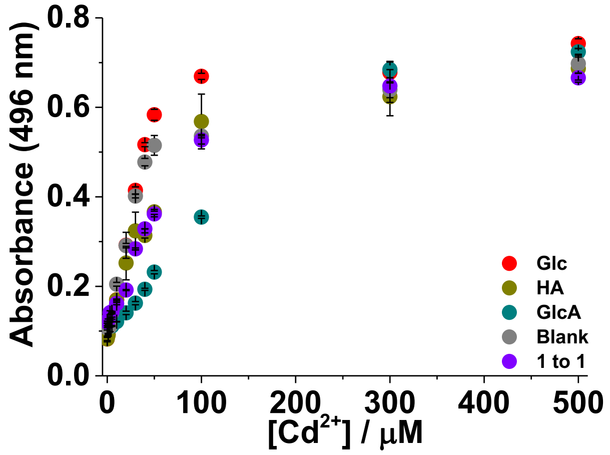

Figure S7. Colorimetric assay of PAR depending on Cd^{2+} concentration. [PAR] = 100 μM, [polymers] = 0.1 mg/ml

Table S1. Calculated slope of lines in Figure 4c, using the "simple linear regression" function in GraphPad Prism.

	C4-Glc	HA	C4-GlcA	1:1
**Slope**	1.0	0.64	0.25	0.47
**Std. Error**	0.019	0.019	0.0062	0.012
**RMSE**	2.4	2.3	1.3	2.6

Table S2. Statistical analysis of Cd^{2+} removal of **1:1 vs. HA** as a function of initial cadmium concentration in Figure 4, which was performed using an unpaired t test.

Concentration	P-value (1:1 vs. HA)
**5**	0.0538
**10**	0.0005
**20**	0.0016
**30**	0.0438
**40**	0.0896
**50**	0.0432

Table S3. Statistical analysis of Cd^{2+} removal of **C4-GlcA vs. HA** (left) and **C4-GlcA vs. 1:1** (right) as a function of initial cadmium concentration in Figure 4, which was performed using an unpaired t test.

Concentration	P-value (C4-GlcA vs. HA)	P-value (C4-GlcA vs. 1:1)
**5**	0.0018	0.0859
**10**	<0.0001	0.0041
**20**	<0.0001	0.0056
**30**	<0.0001	<0.0001
**40**	<0.0001	<0.0001
**50**	<0.0001	<0.0001

ICP-MS Sample Preparation and Analysis

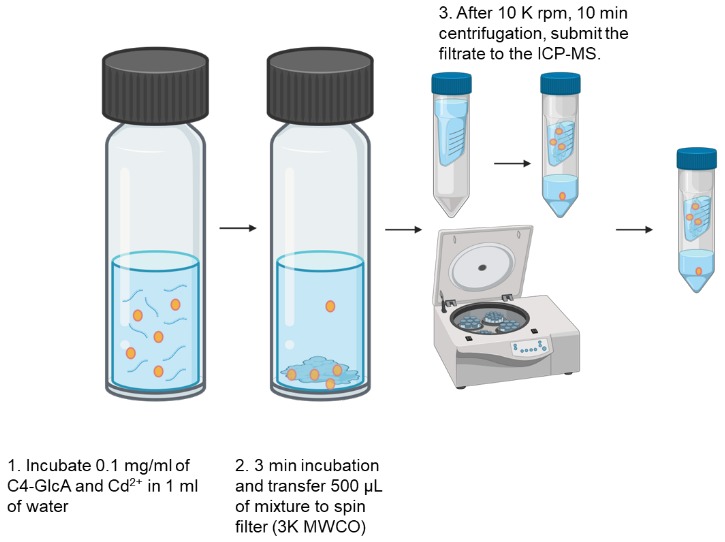

Scheme S7. Sample preparation procedure for ICP-MS analysis of heavy metal sequestration.

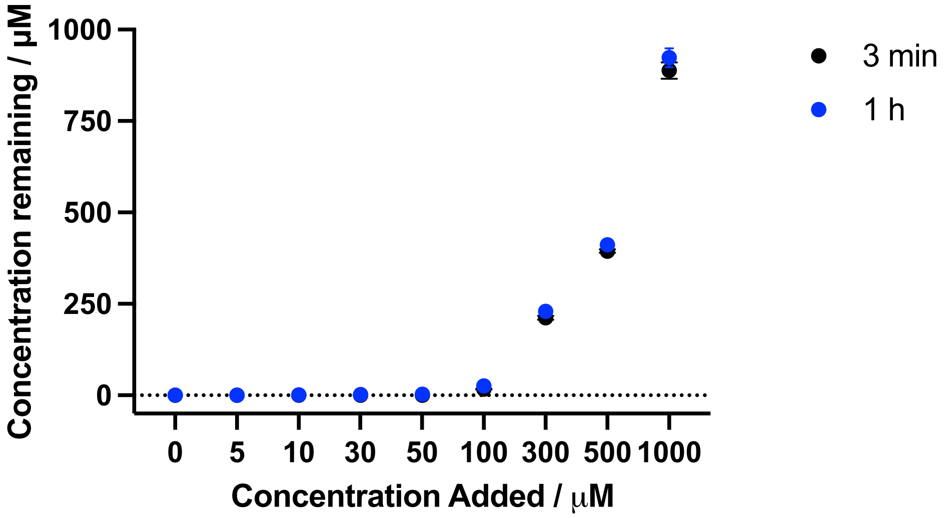

Figure S8. Amount of Cd^{2+} remaining by **C4-GlcA**, as determined by ICP-MS, at increasing concentration of Cd^{2+}. [**C4-GlcA**] = 0.1 mg/ml. Black = 3 min incubation, Blue = 1 h incubation.

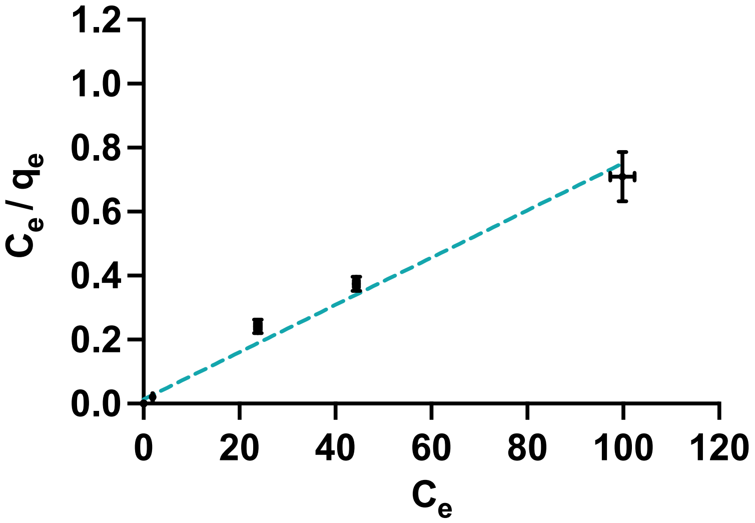

	Ce/qe
Best-fit values	
Slope	0.007386
Y-intercept	0.01312
X-intercept	-1.776
1/slope	135.4

	Ce/qe
R squared	0.9779

Figure S9. Linearized Langmuir isotherm of Cd^{2+} binding by **C4-GlcA**.

TEM Sample Preparation

TEM images were acquired using a JEOL NEOARM Low kV STEM Corrected (200 kV) instrument at the Texas Materials Institute. Carbon filmcoated 400-mesh copper grids (Ted Pella, Inc) were treated for 60 s at 10 mA on a glow discharge unit, after which a solution (5 μL) of each test sample (1 mg/mL in H_2O) was dropped onto the grid and allowed to set for 60 s before wicking away excess solution. The samples were then negatively stained by dropping 5 μL of 2 wt% uranyl acetate onto the grid, allowed to sit for 60 s, and excess solution was wicked away using the edge of filter paper. The negative staining process was carried out twice per sample. The samples were allowed to fully dry (ca. 3 h) before analysis.

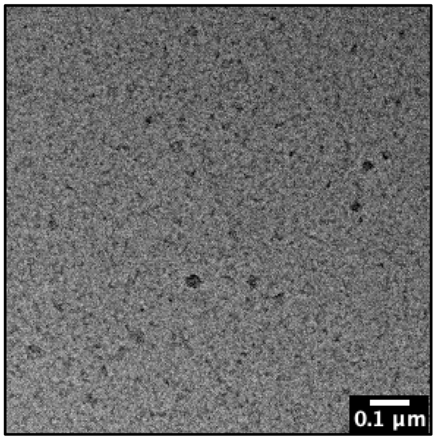

Figure S10. TEM image of acidified **C4-GlcA**. Sample preparation was as described above with the following exception: the sample solution was acidified to pH = 2 with HCl.

Real Lake Water Sample Preparation

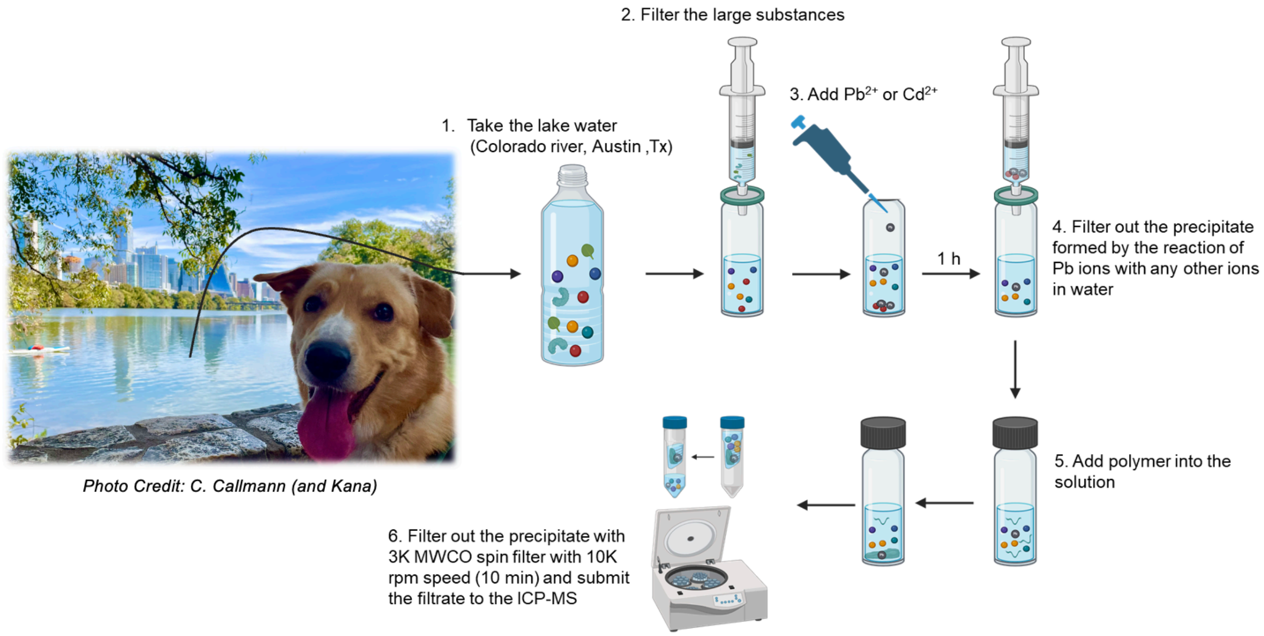

Scheme S8. Sample preparation of lake water sample for ICP-MS analysis of heavy metal sequestration. Reaction volume = 1 mL, [**C4-GlcA**] = 0.1 mg/mL. Added concentration of heavy metals = 100 μM.

Procedure for pH-Dependent Capture-and-Release

Reversibility data were obtained using the Amicon Ultra-4 spin filter system. Initially, 0.1 mg of **C4-GlcA** was placed in the spin filter, followed by the addition of 1 mL of 100 μM Cd^{2+} solution. After centrifuging for 15 minutes, the filtrate was collected and sent for analysis using ICP-MS. Subsequently, 1 mL of 0.01 M HCl solution was added to the spin filter and centrifuged again, with the resulting filtrate also being sent for ICP-MS analysis. To neutralize the solution, 1 ml of pH 8 Tris-HCl buffer was added to the spin filter, which was then washed twice with water. This cycle was considered one round, and a total of four cycles were conducted to evaluate the stability and reversibility of **C4-GlcA**. A video of this process can be seen as a separate SI file associated with the manuscript.

^{1}H-NMR and ^{13}C-NMR Spectra

^{1}H-NMR Spectrum of **C4-OH** in $CDCl_3$ (500 MHz):

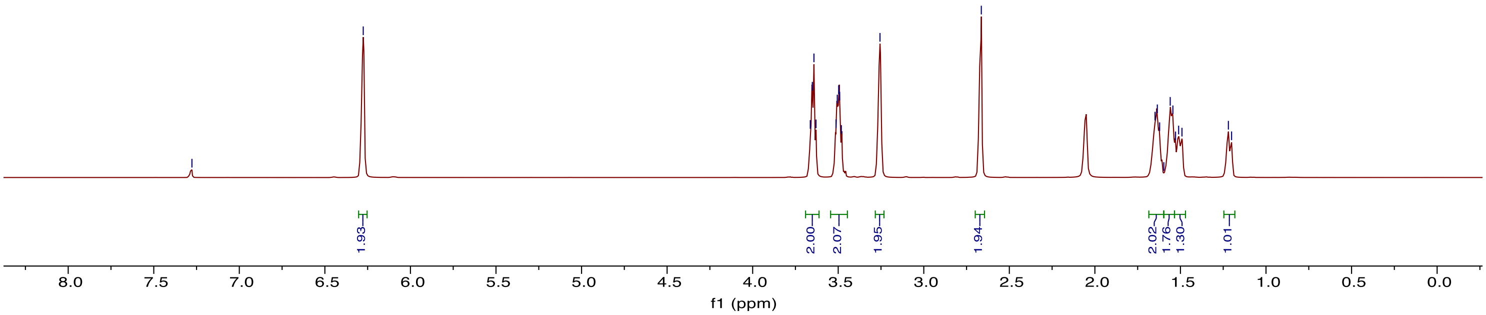

^{13}C-NMR Spectrum of **C4-OH** in $CDCl_3$ (126 MHz):

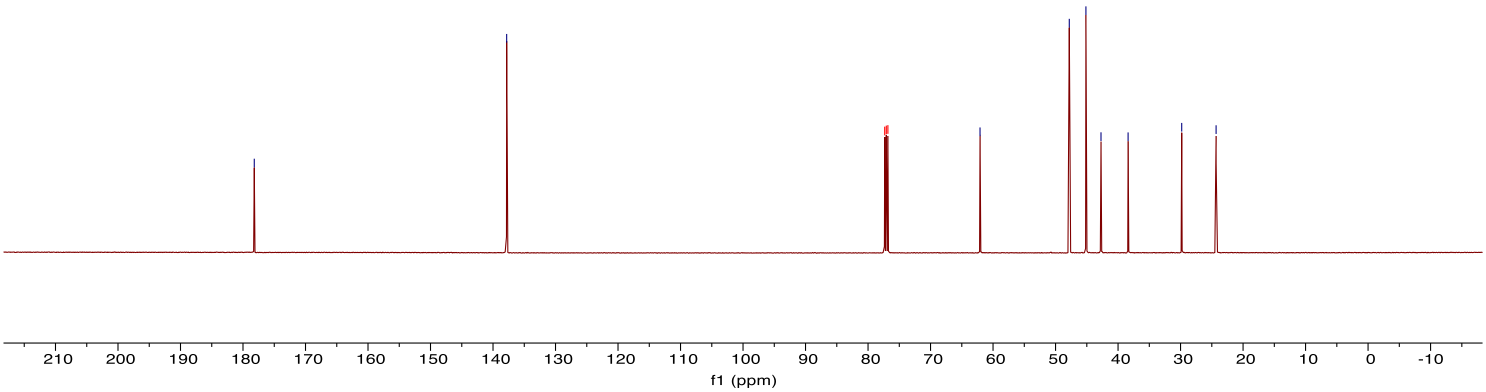

^{1}H-NMR Spectrum of **1** in $CDCl_3$ (400 MHz)

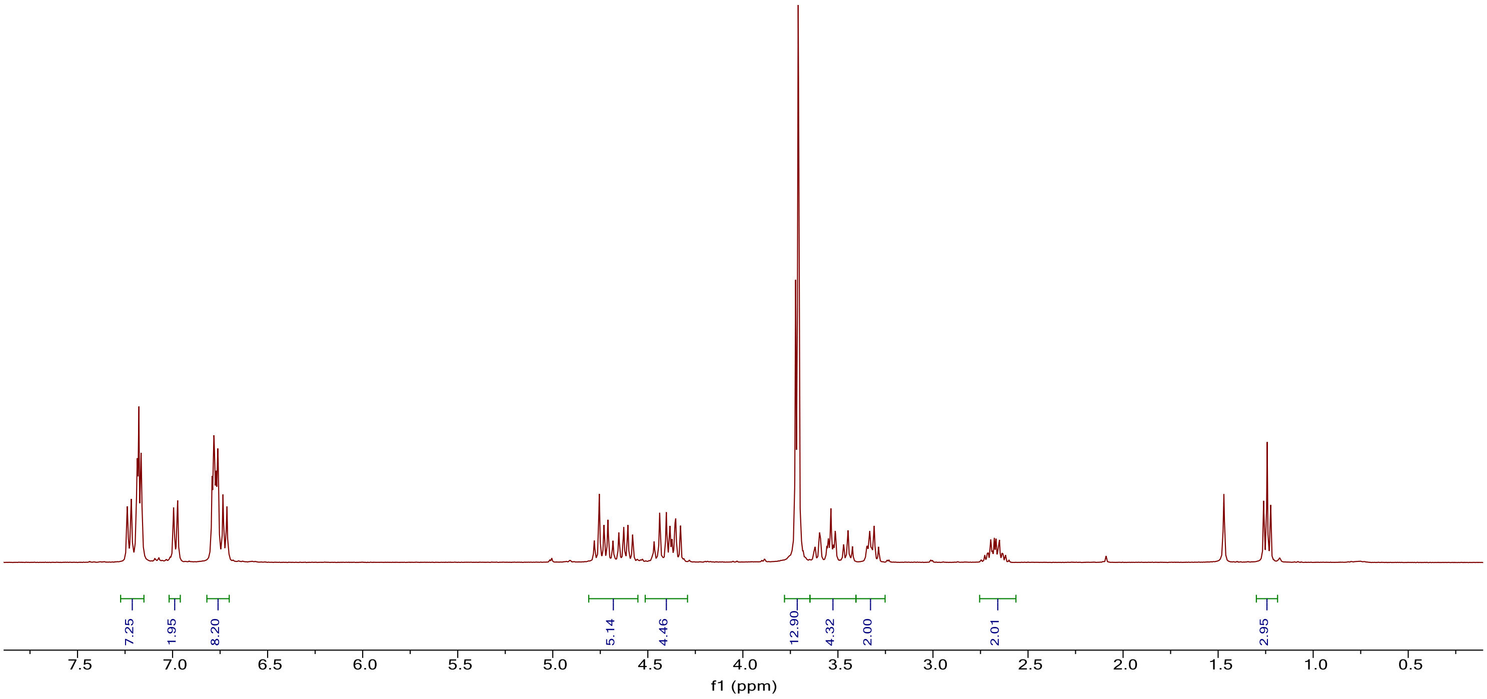

^{13}C-NMR Spectrum of **1** in $CDCl_3$ (100 MHz):

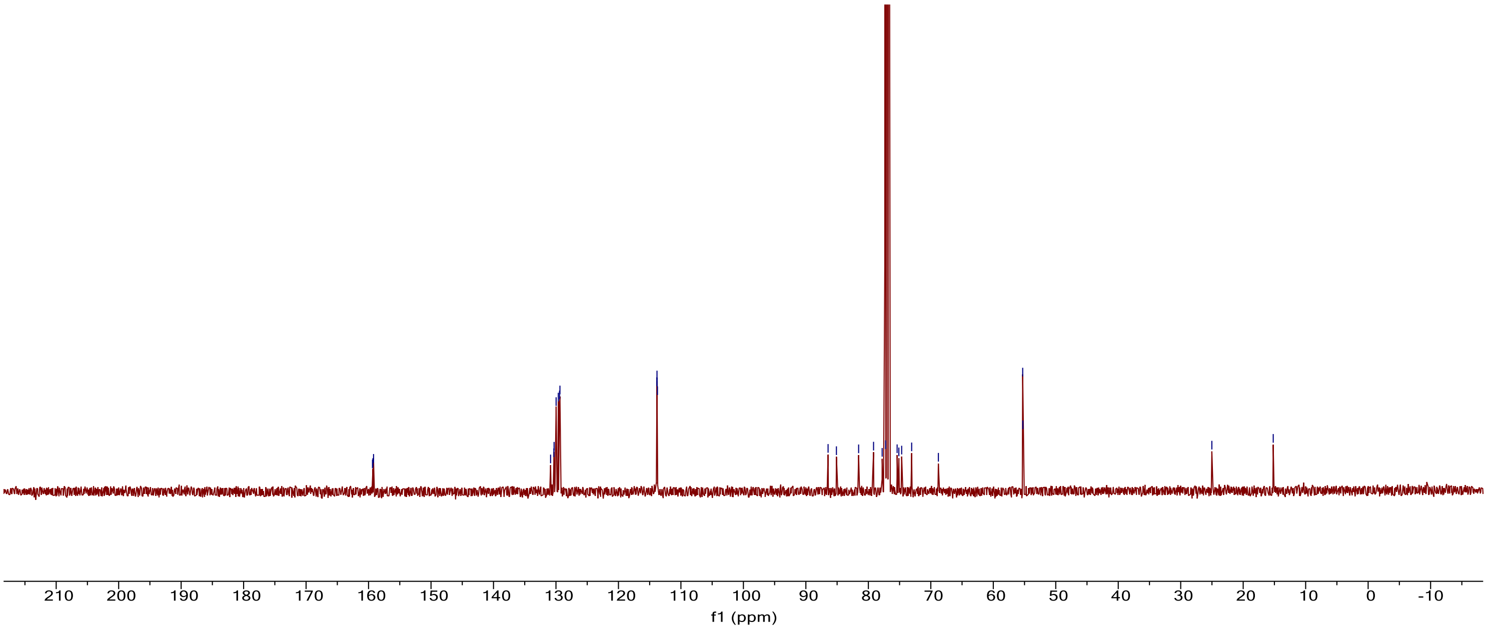

^{1}H-NMR Spectrum of **2** in $CDCl_3$ (400 MHz):

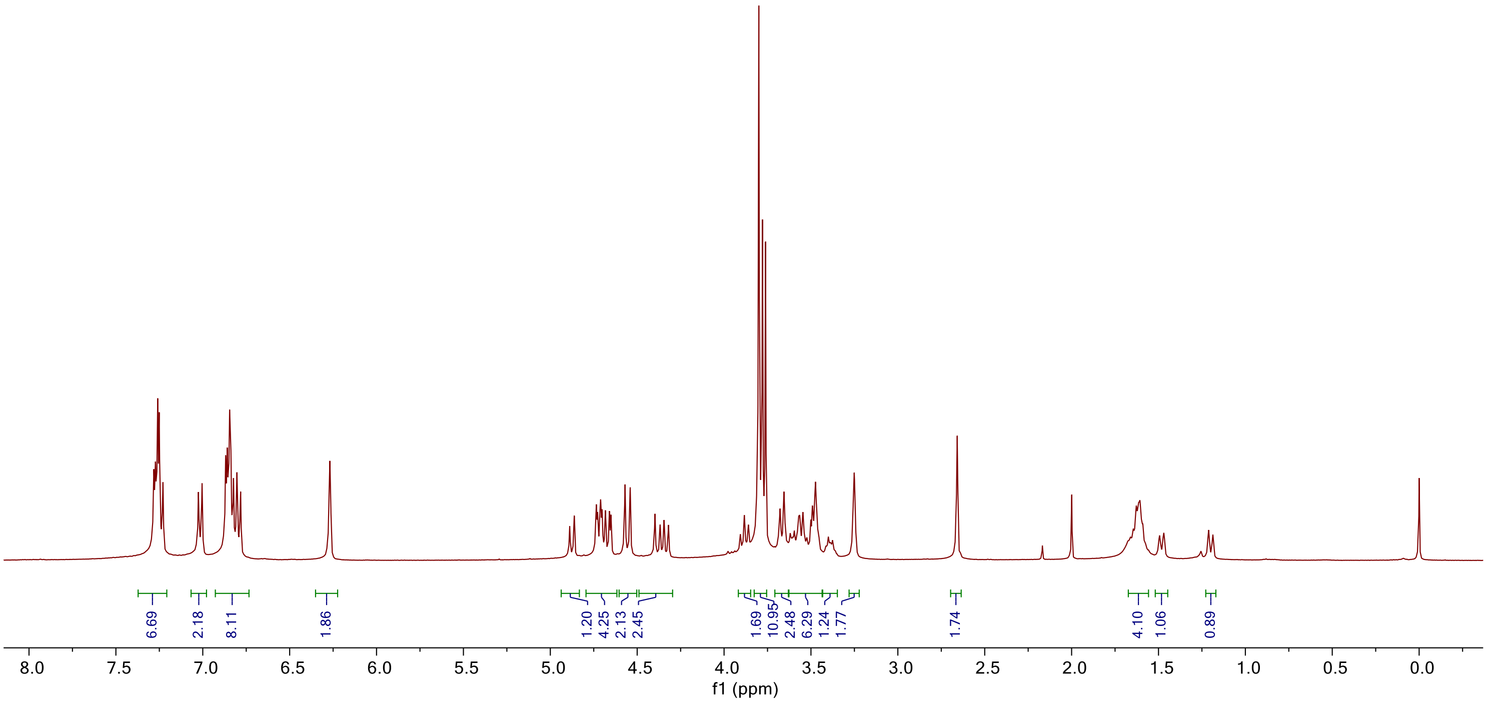

^{13}C-NMR Spectrum of **2** in $CDCl_3$ (100 MHz):

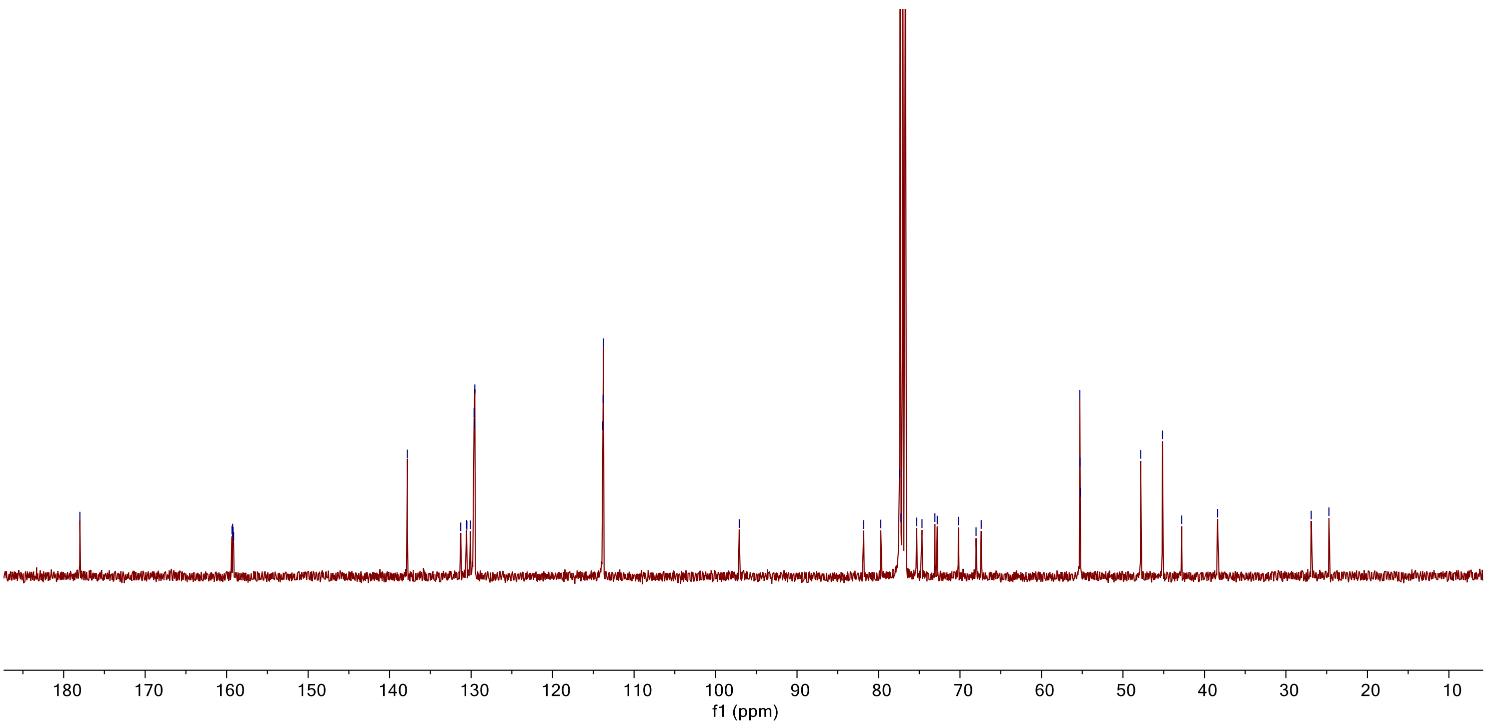

2D-HSQC Spectrum of **2** in $CDCl_3$:

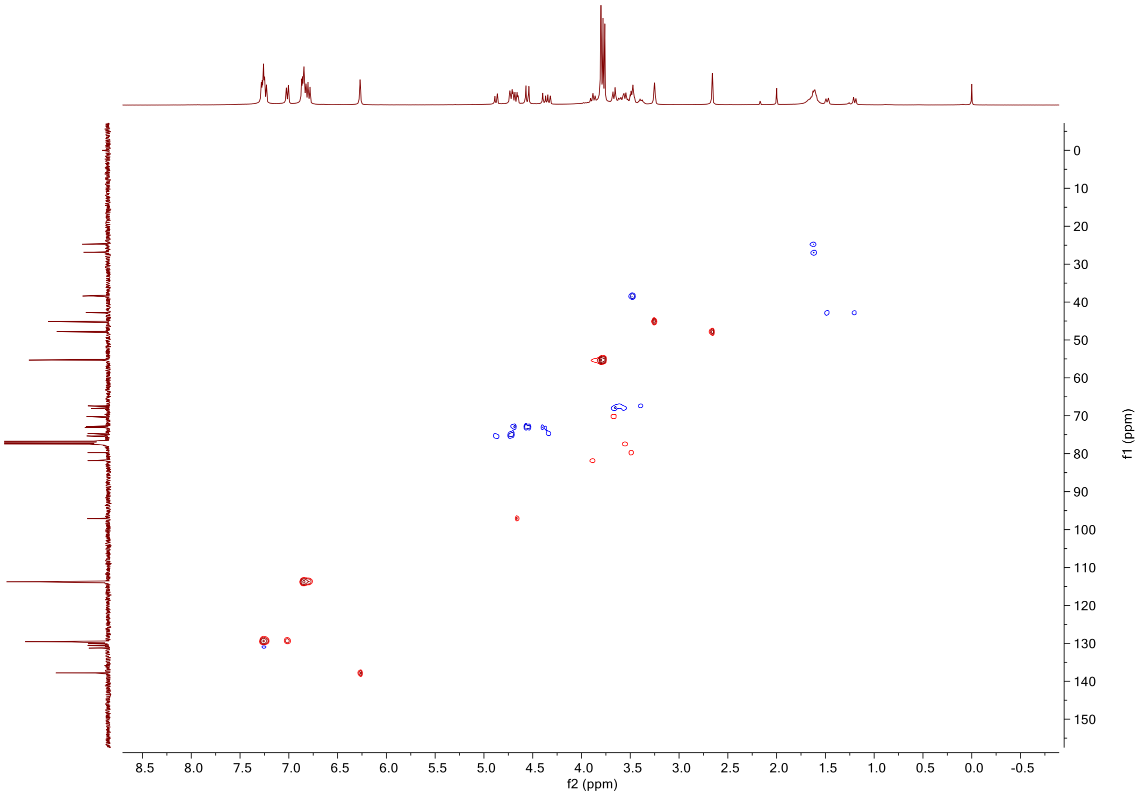

2D-COSY Spectrum of **2** in $CDCl_3$:

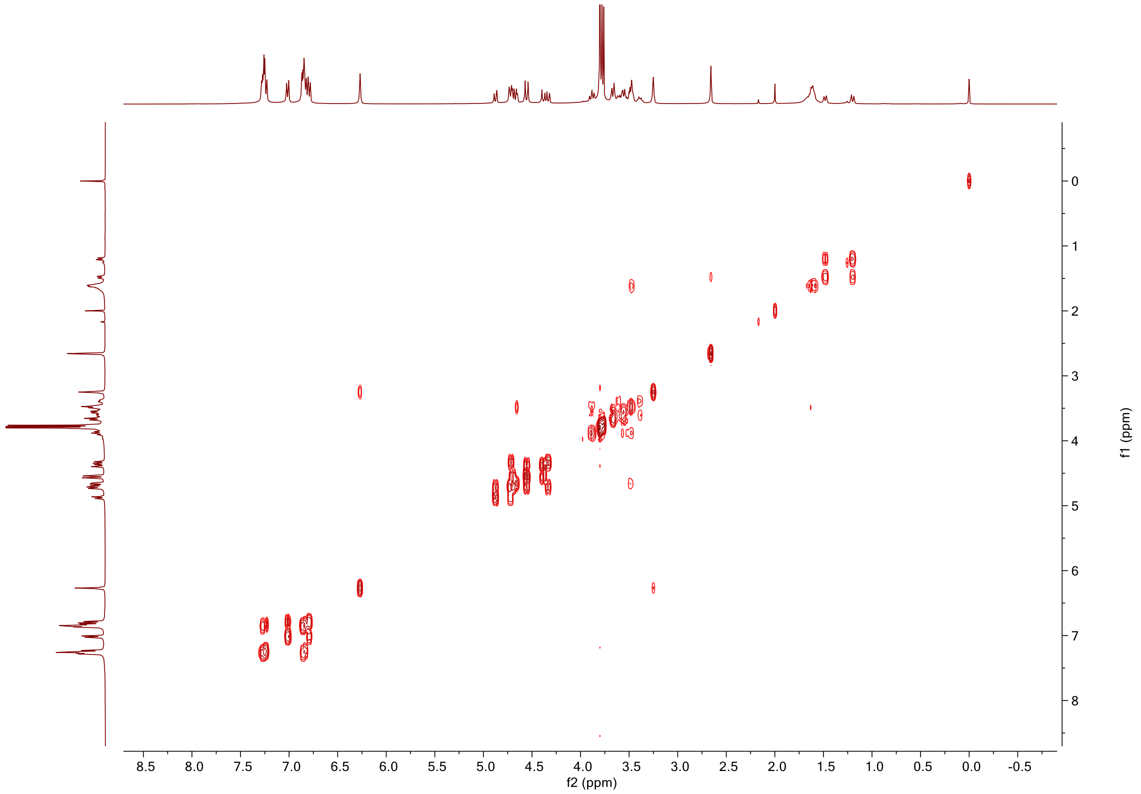

^{1}H-NMR Spectrum of **mono-C4-Glc** in MeOD (400 MHz):

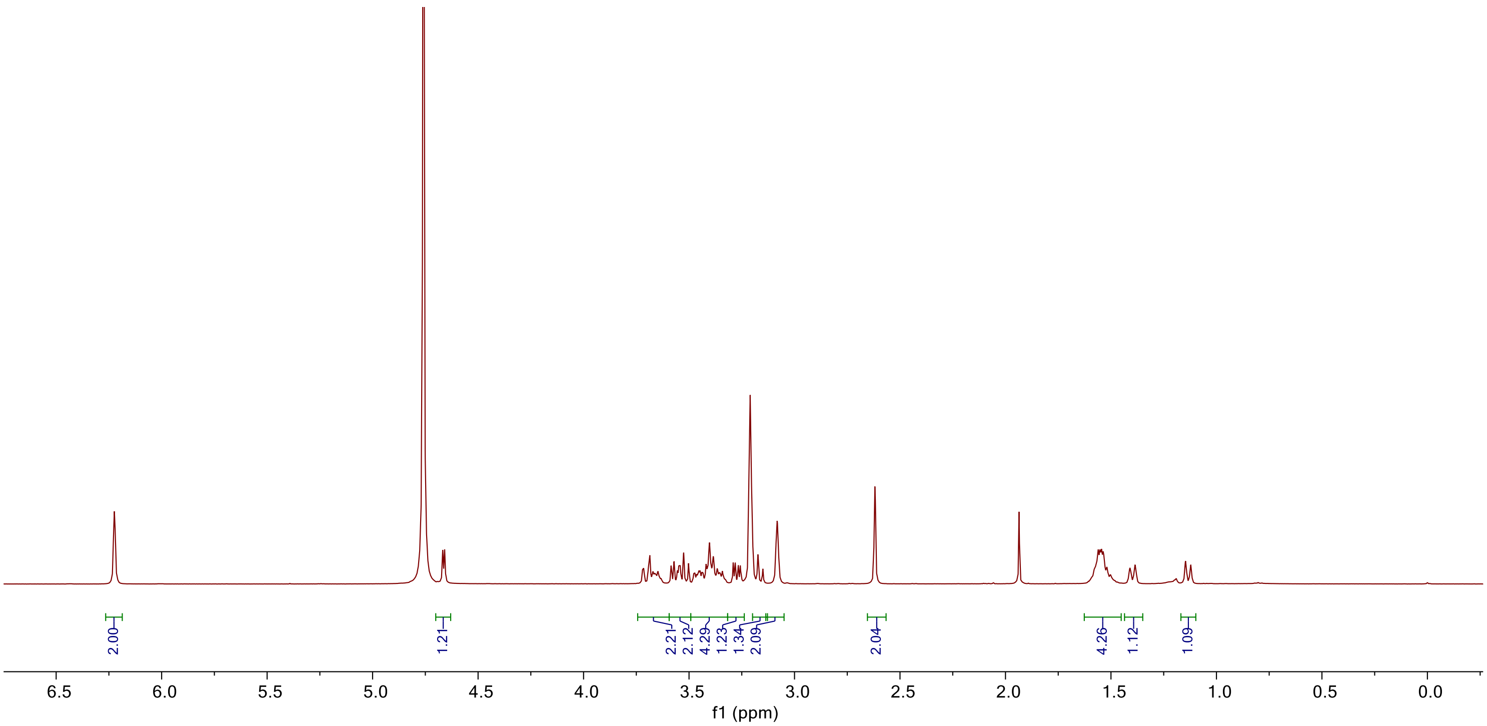

^{13}C-NMR Spectrum of **mono-C4-Glc** in MeOD (100 MHz):

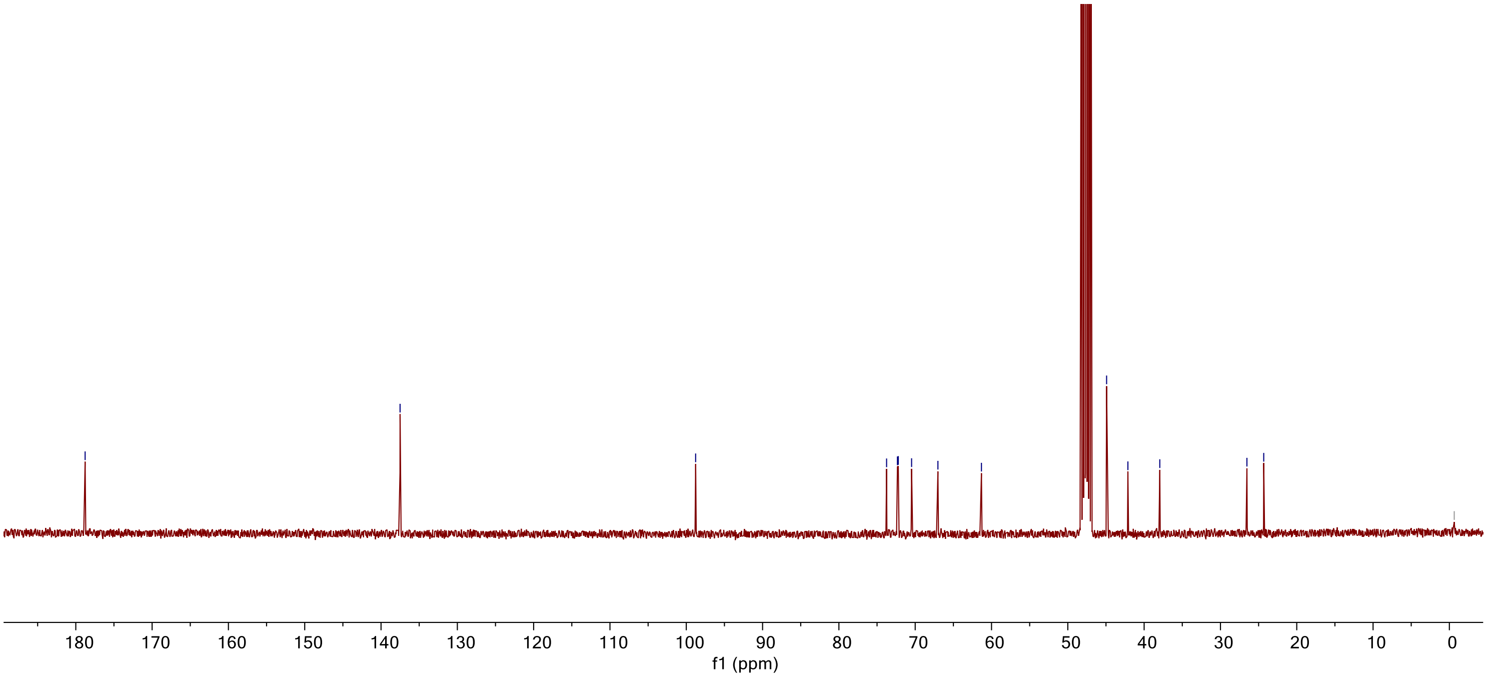

2D-HSQC Spectrum of **mono-C4-Glc** in MeOD:

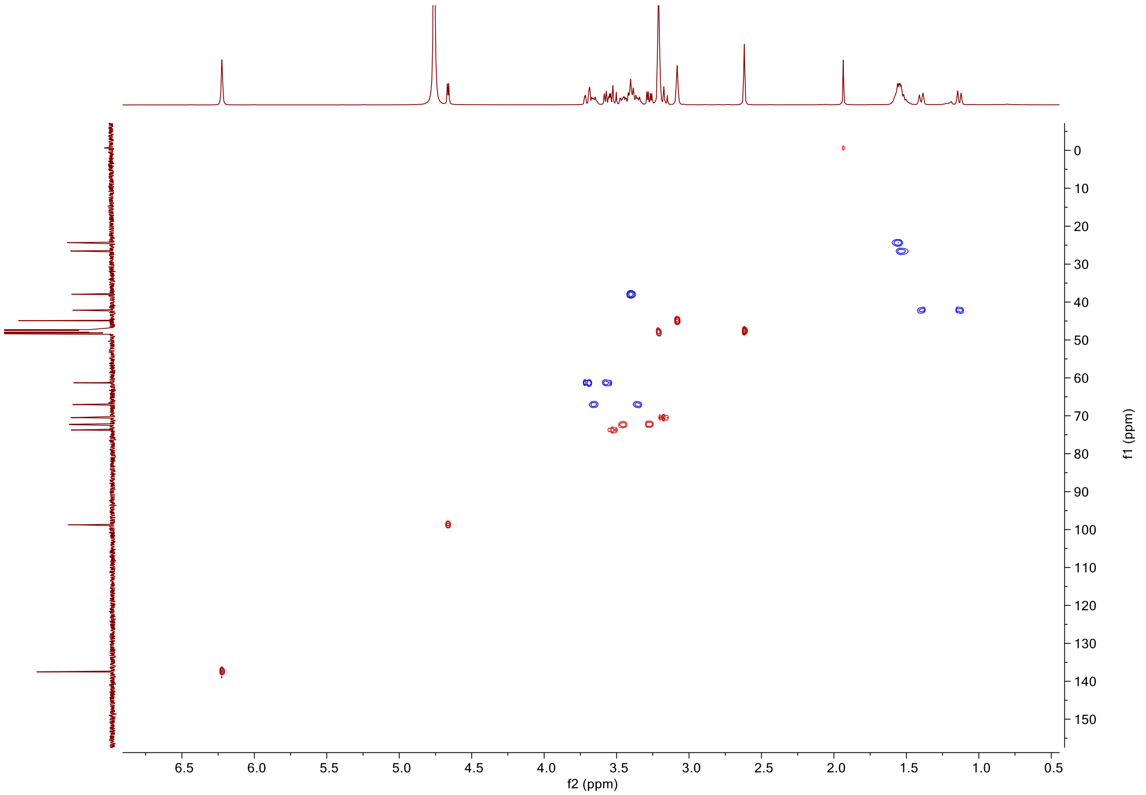

2D-COSY Spectrum of **C4-Glc** in MeOD:

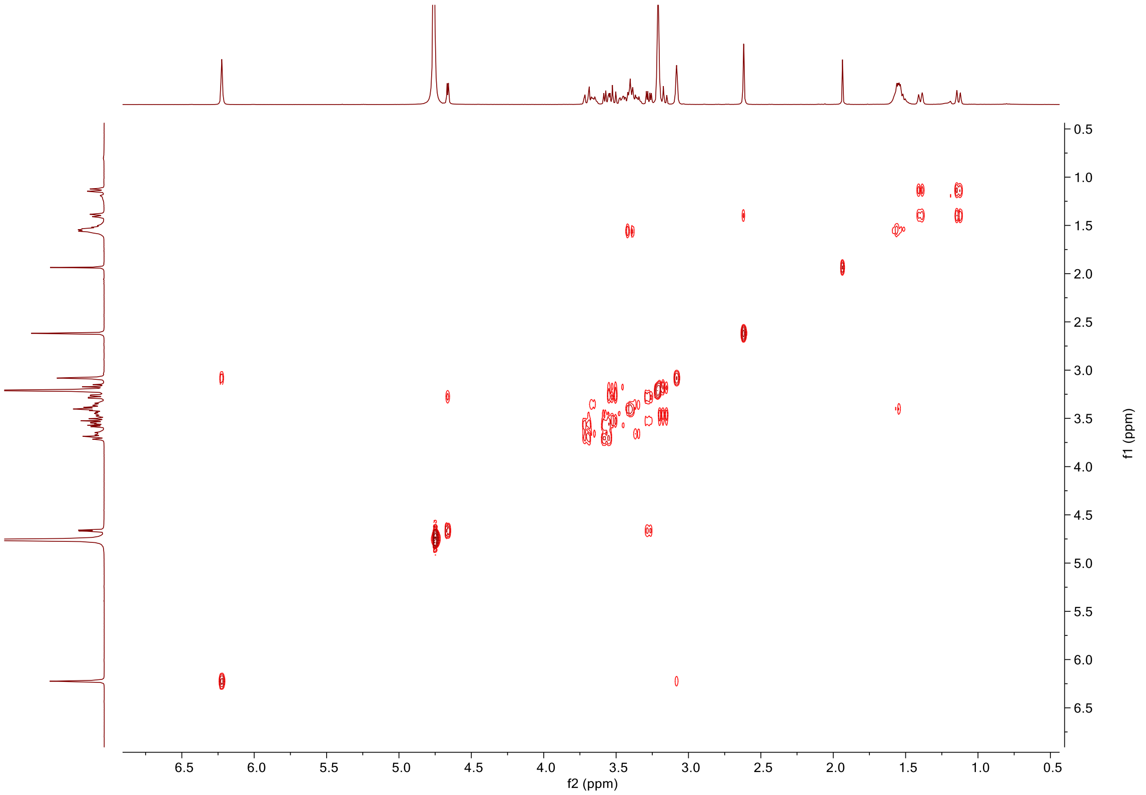

^{1}H-NMR Spectrum of **4** in $CDCl_3$ (400 MHz):

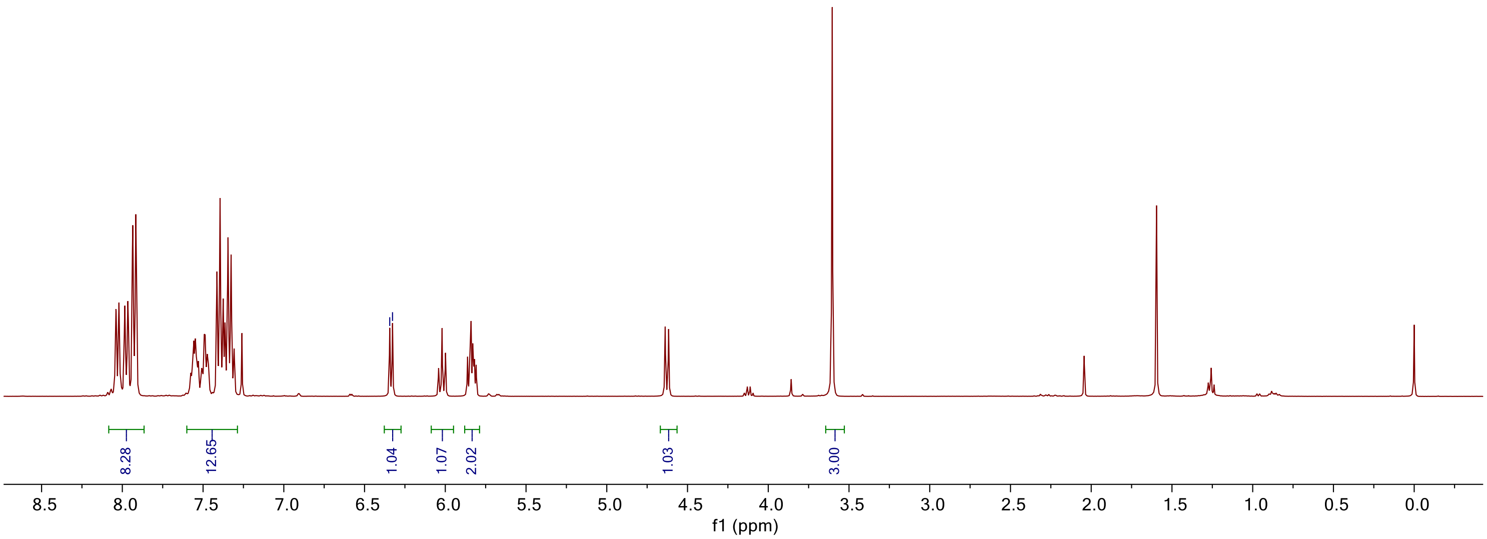

^{13}C-NMR Spectrum of **4** in $CDCl_3$ (100 MHz):

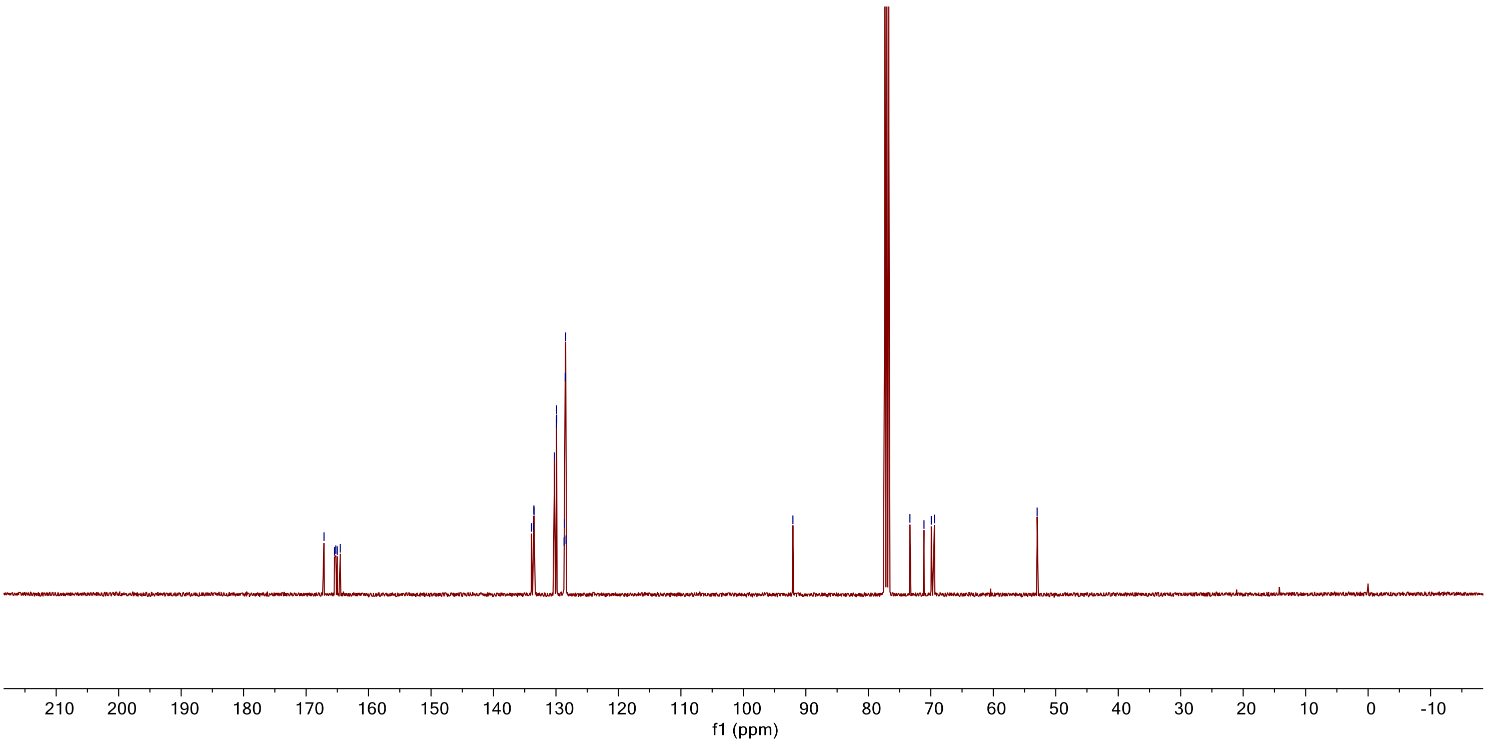

2D-HSQC Spectrum of **4** in $CDCl_3$:

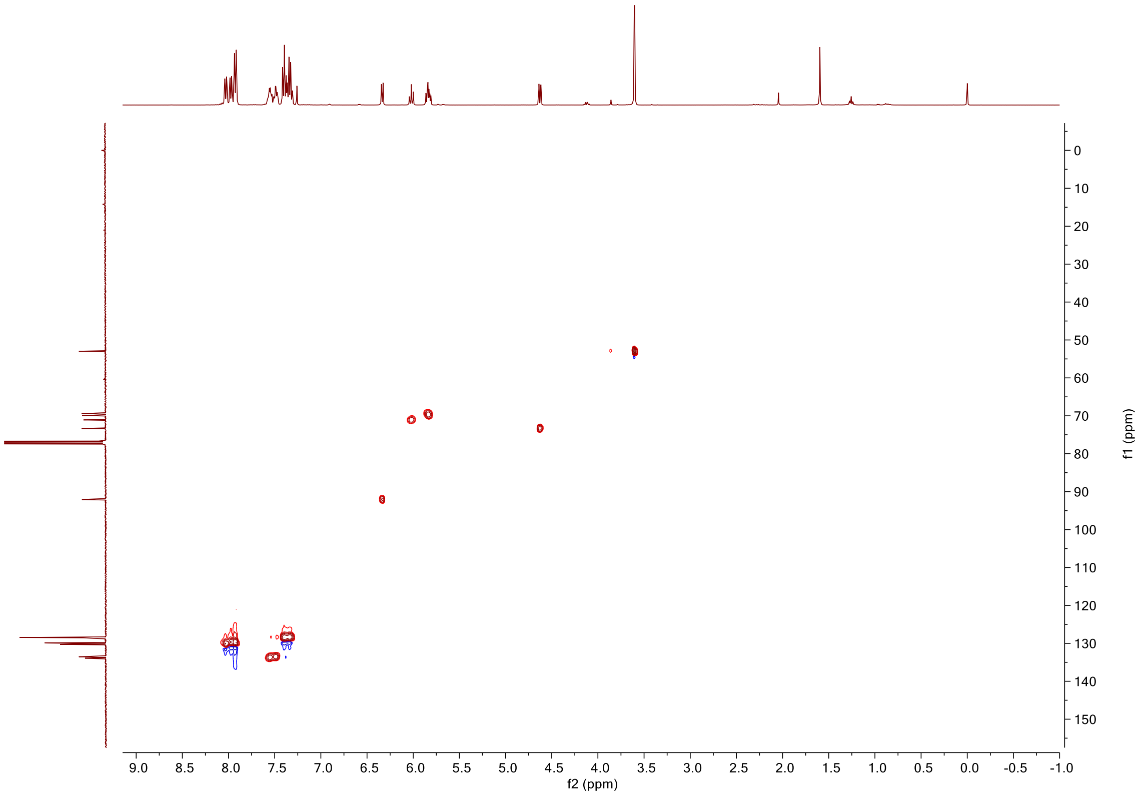

2D-COSY Spectrum of **4** in $CDCl_3$:

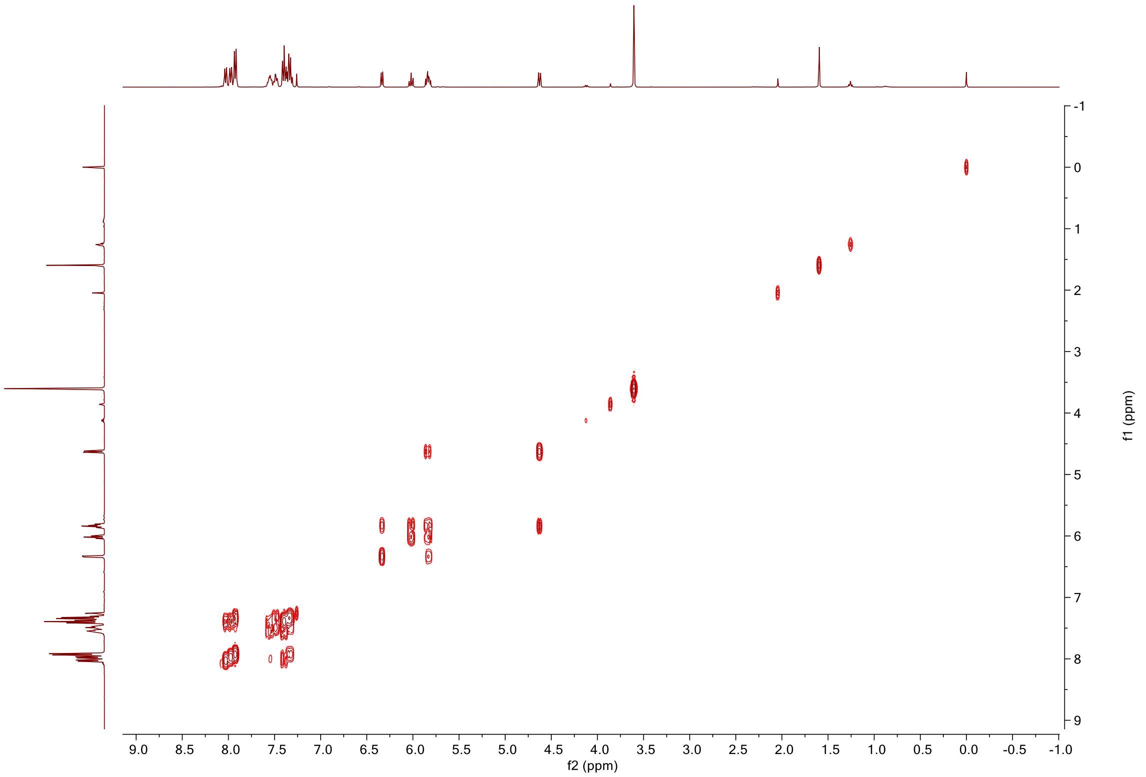

^{1}H-NMR Spectrum of **5** in $CDCl_3$ (400 MHz):

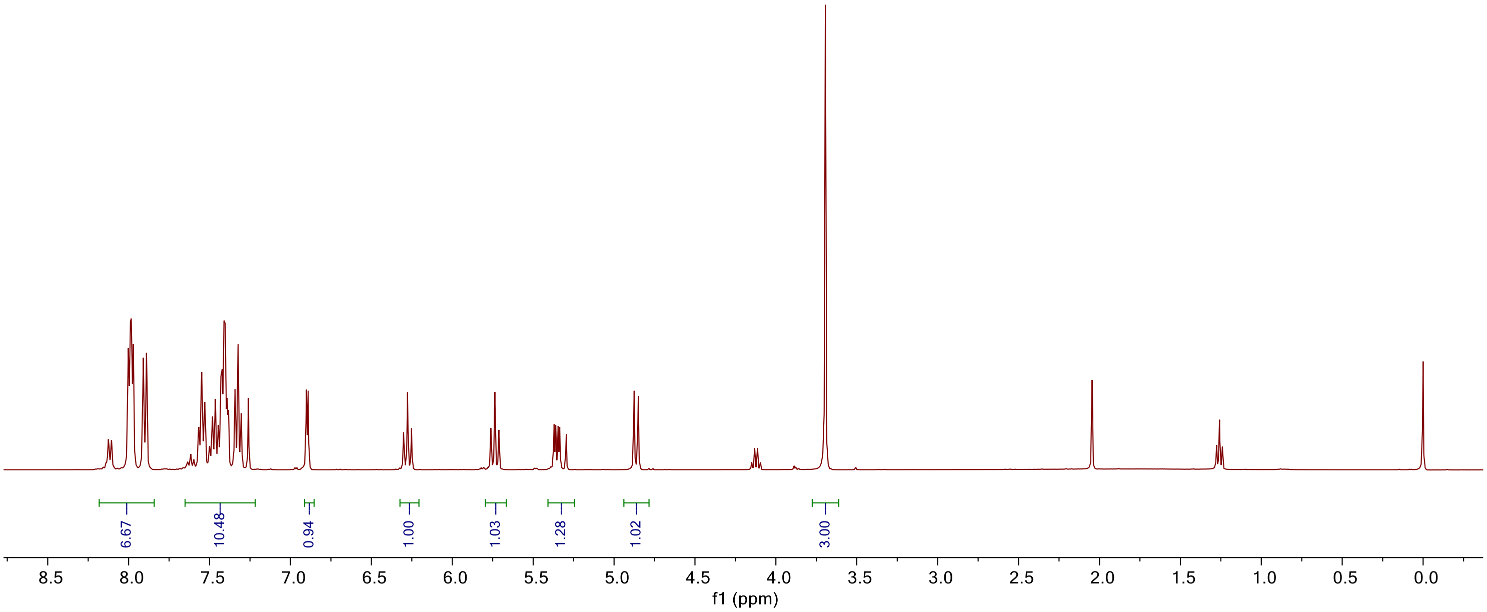

^{13}C-NMR Spectrum of **5** in $CDCl_3$ (100 MHz):

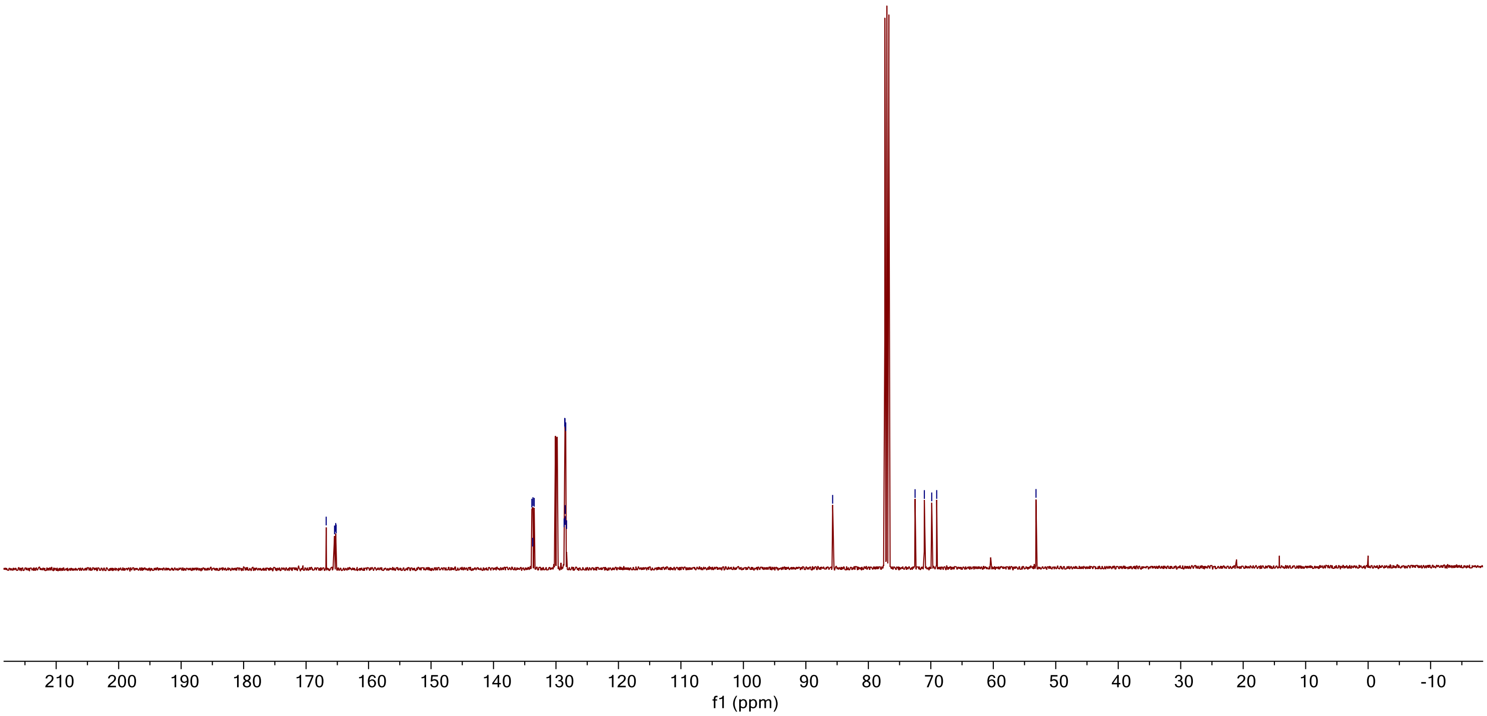

2D-HSQC Spectrum of **5** in $CDCl_3$:

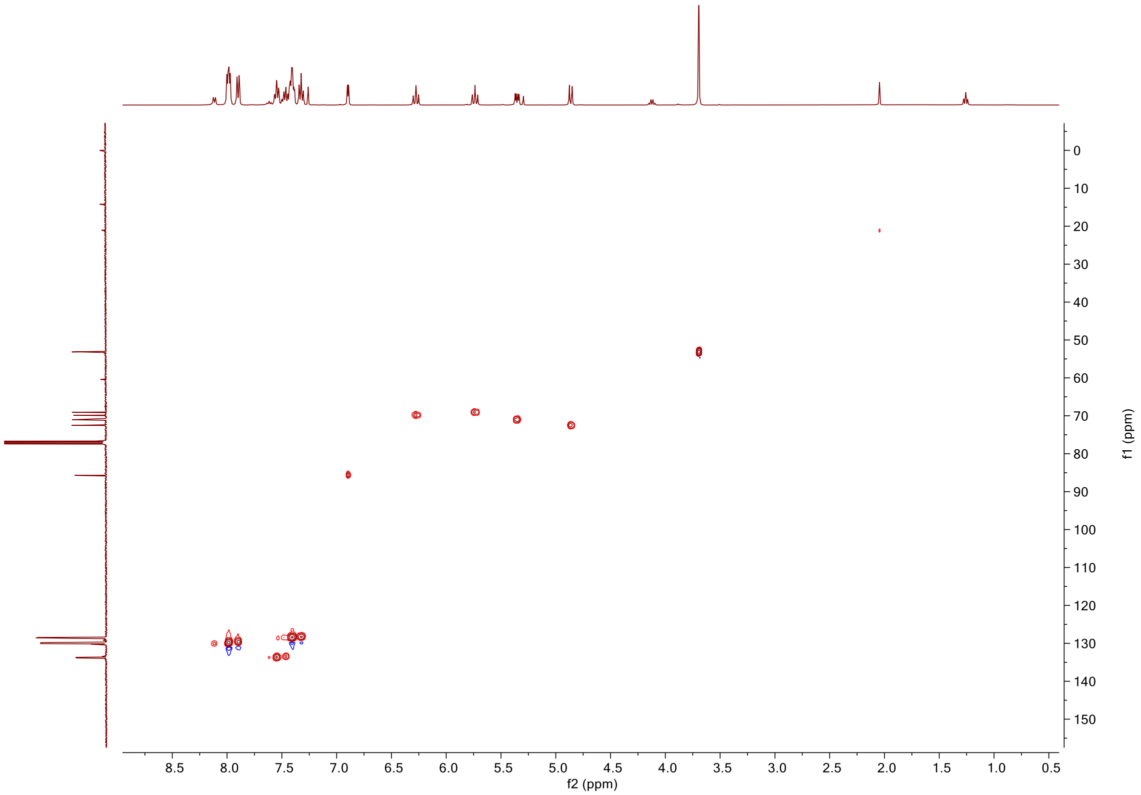

2D-COSY Spectrum of **5** in $CDCl_3$:

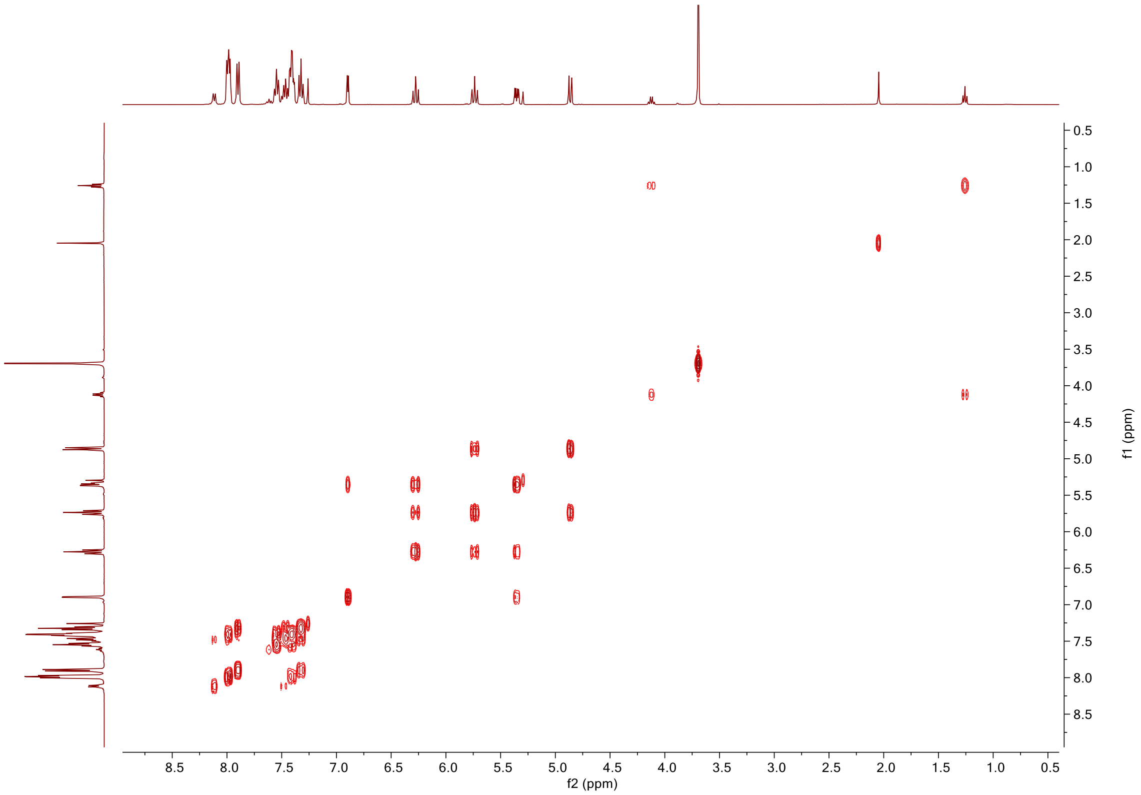

^{1}H-NMR Spectrum of **6** in $CDCl_3$ (400 MHz):

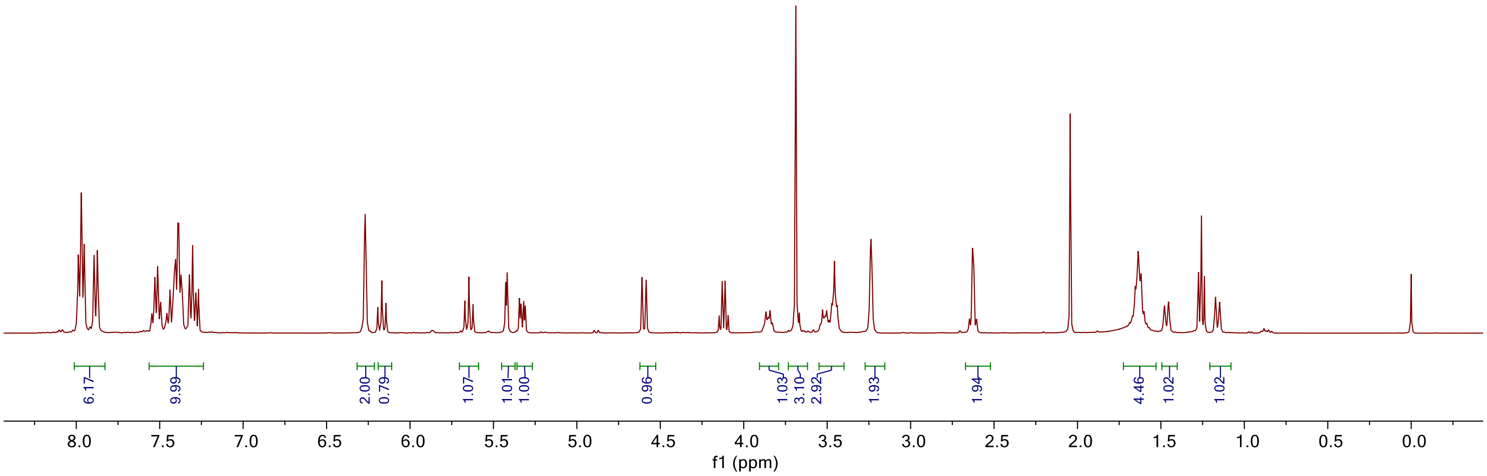

^{13}C-NMR Spectrum of **6** in $CDCl_3$ (100 MHz):

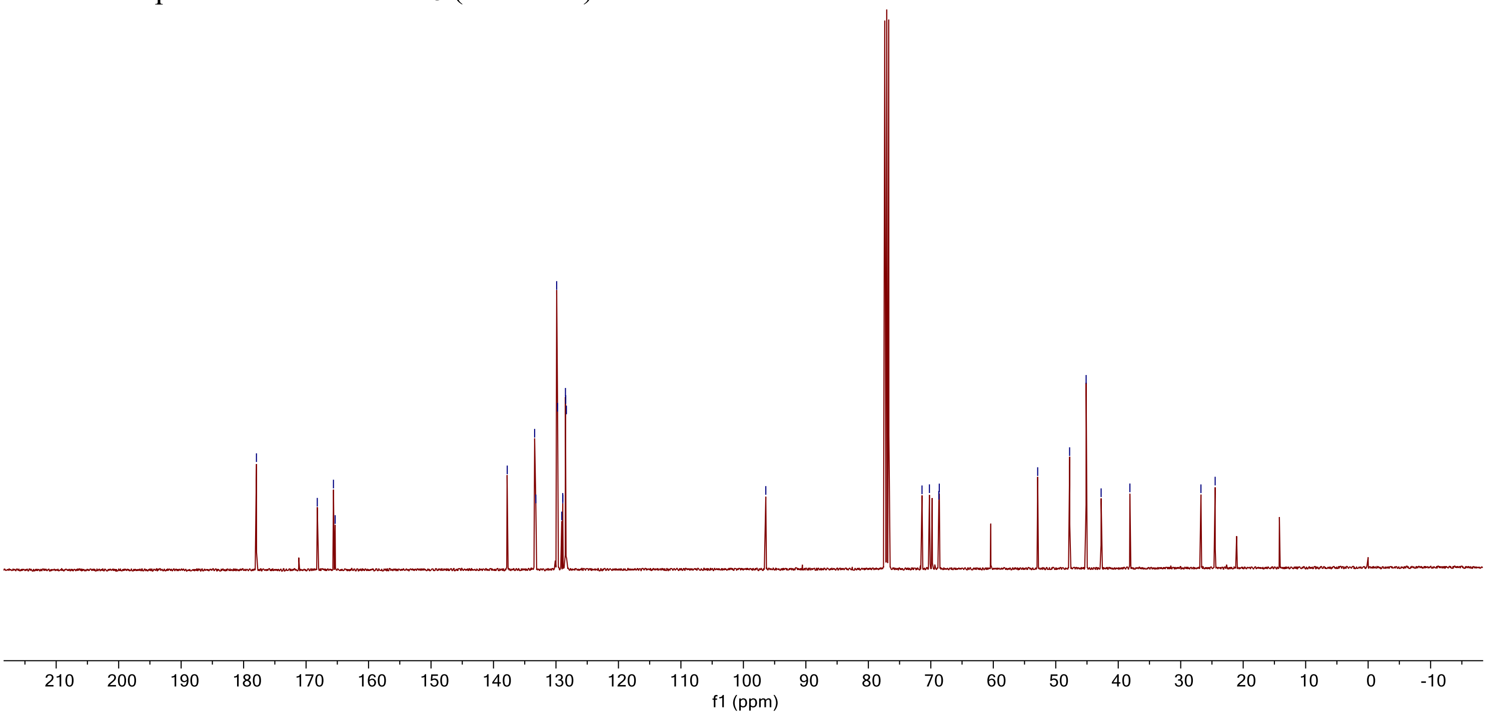

2D-HSQC Spectrum of **6** in $CDCl_3$:

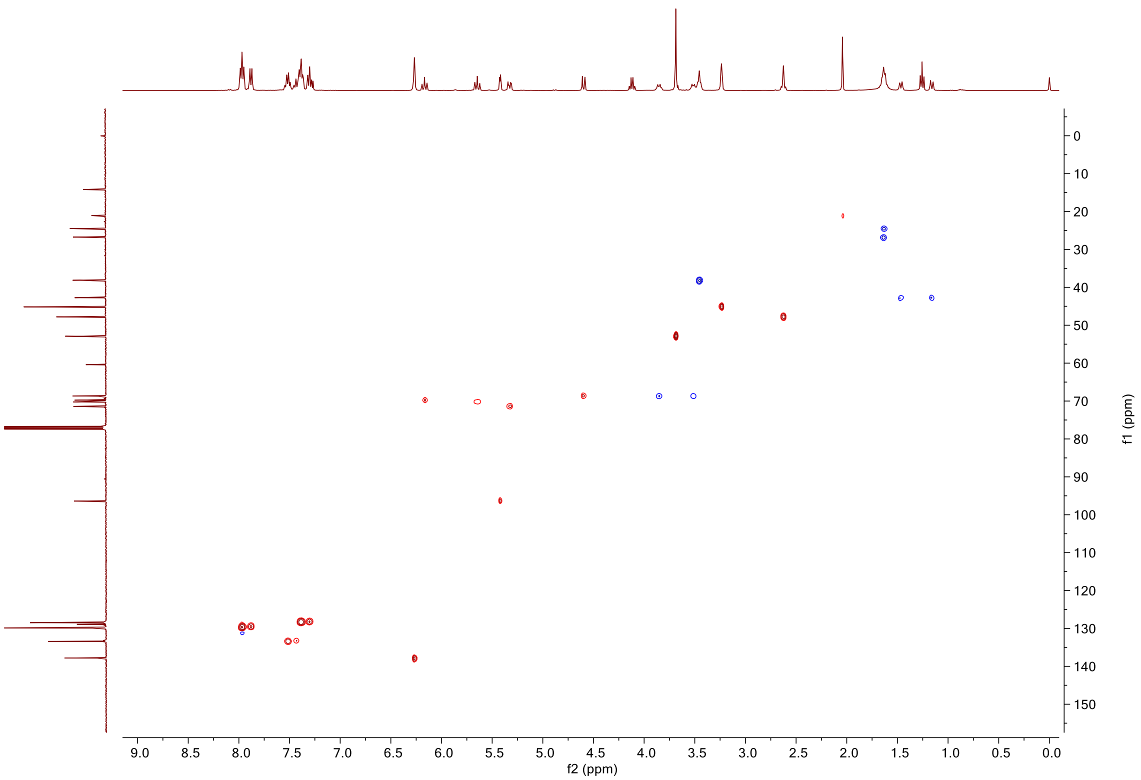

2D-COSY Spectrum of **6** in $CDCl_3$:

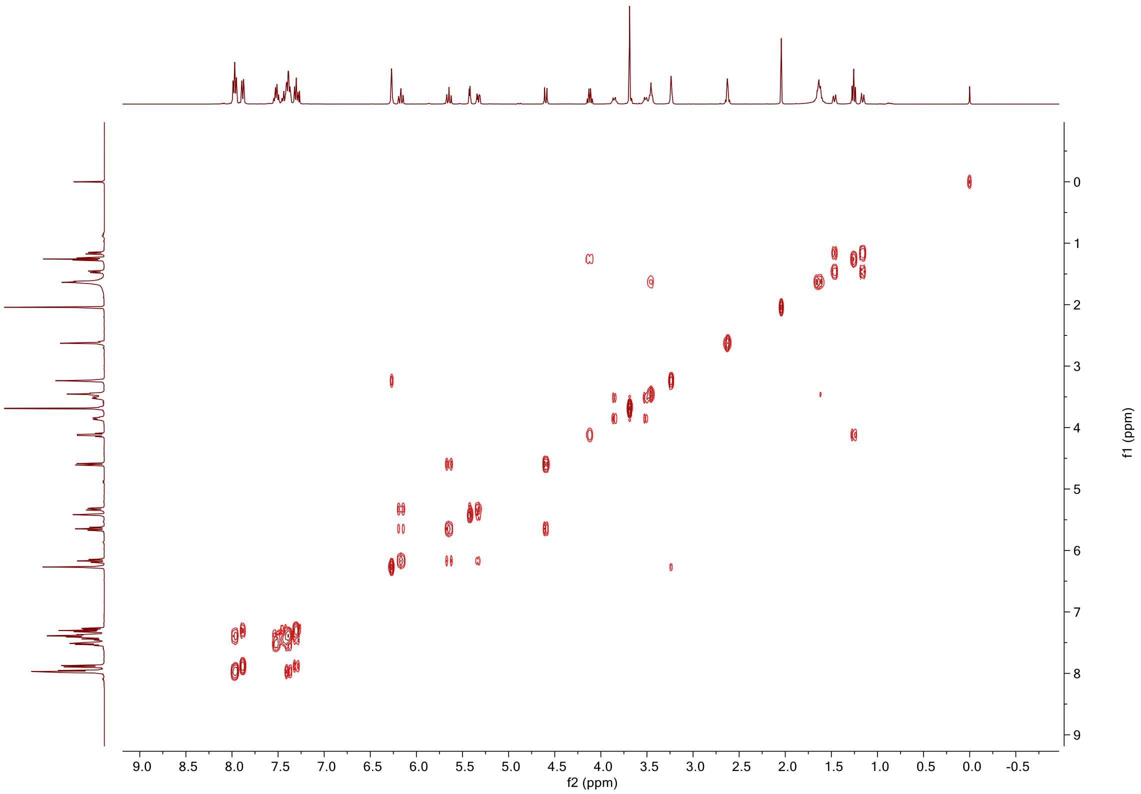

^{1}H-NMR Spectrum of **mono-C4-GlcA-Me** in MeOD (400 MHz):

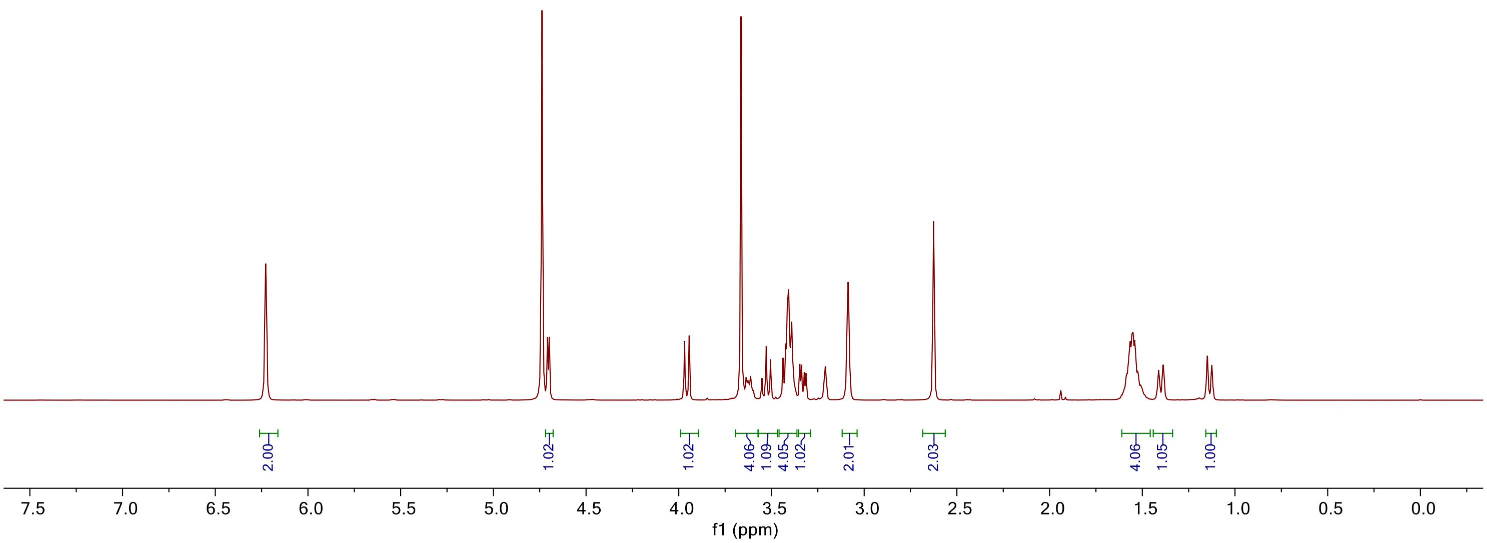

^{13}C-NMR Spectrum of **mono-C4-GlcA-Me** in MeOD (100 MHz):

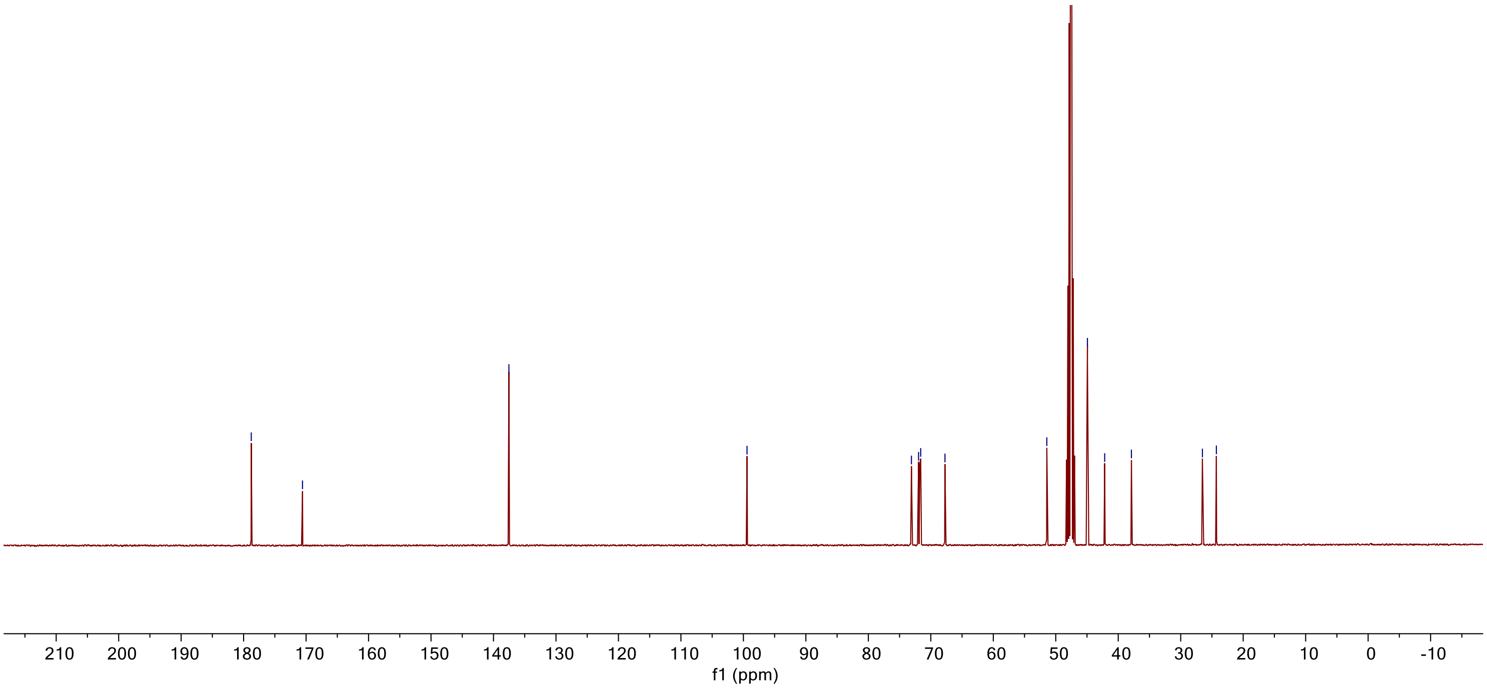

2D-HSQC Spectrum of **mono-C4-GlcA-Me** in MeOD:

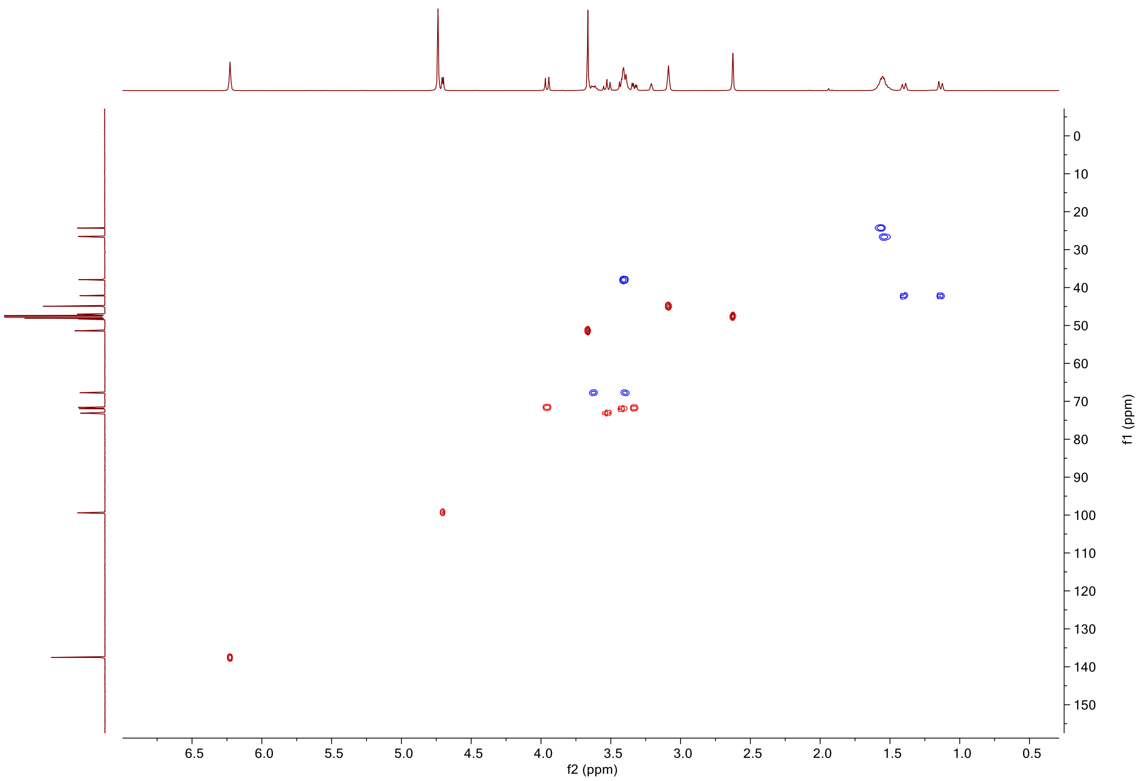

2D-COSY Spectrum of **mono-C4-GlcA-Me** in MeOD:

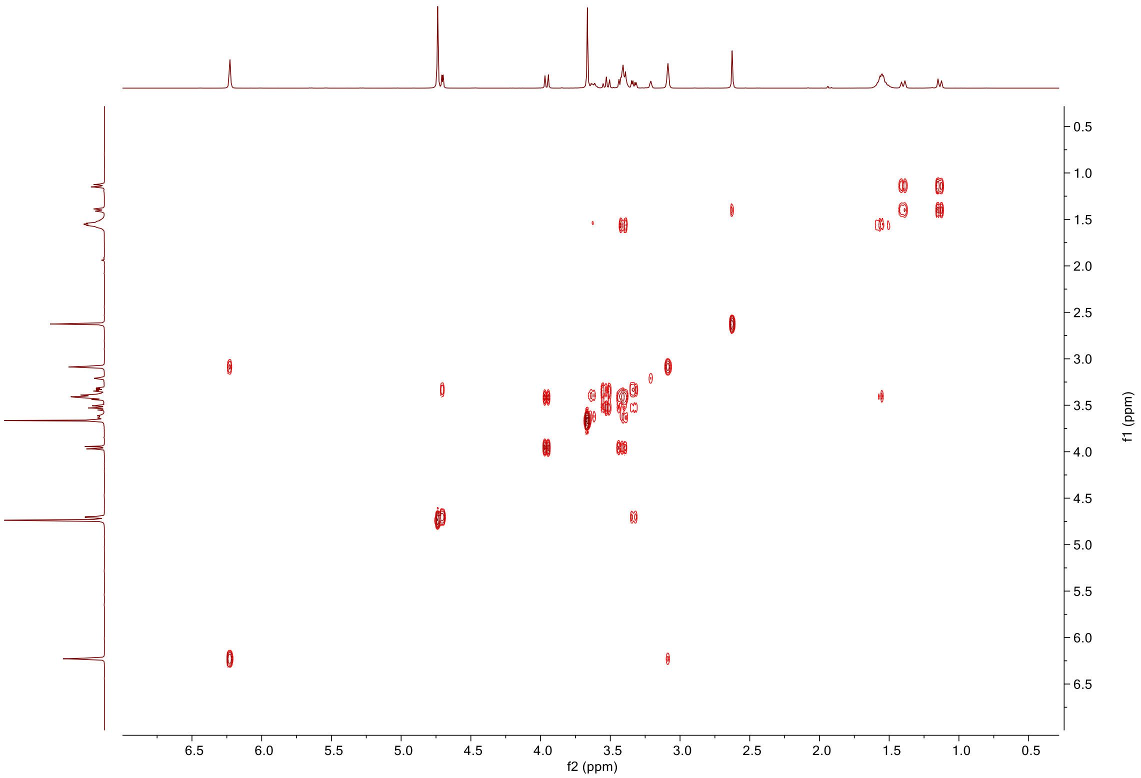

^{1}H-NMR Spectrum of **mono-C4-GlcA** in Acetonitrile-d_3 (400 MHz):

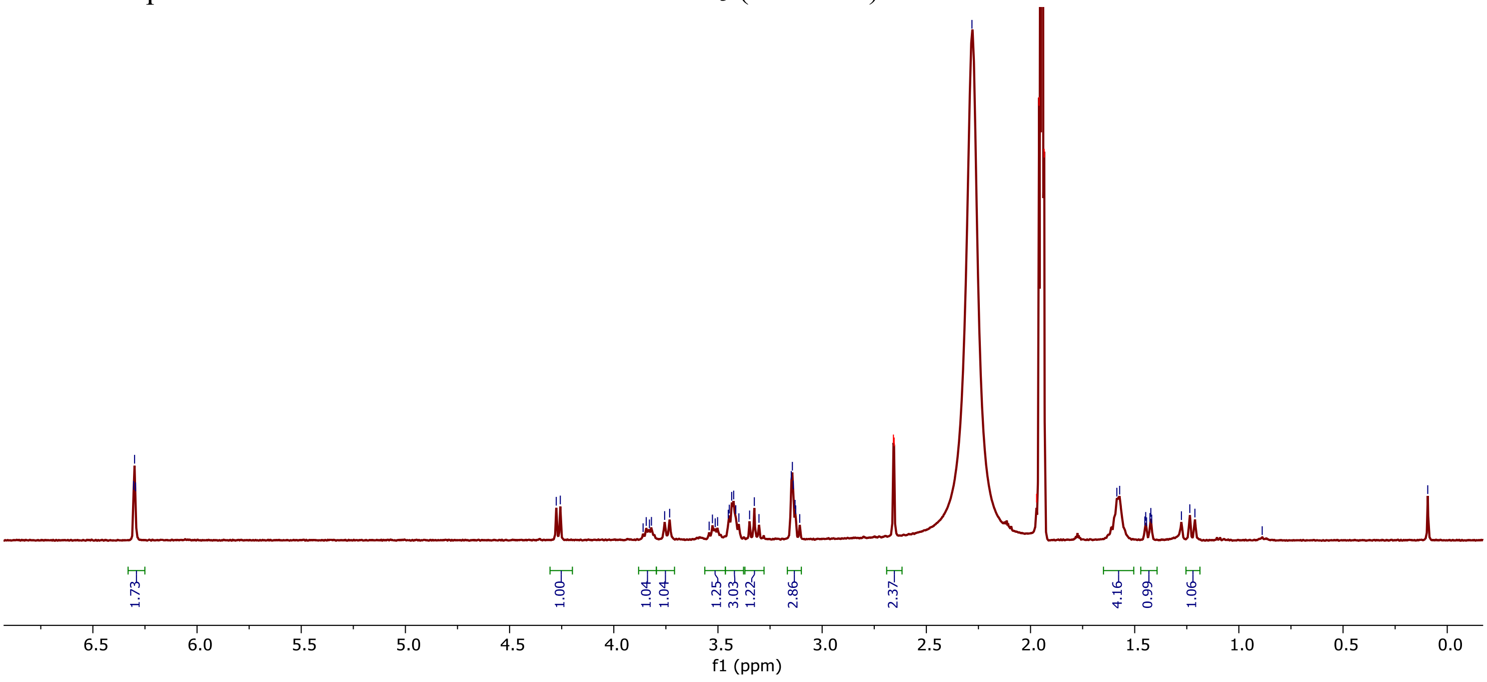

Supplement: Supplementary file 2 — oc4c01010_si_002.pdf [file oc4c01010_si_002.pdf]
